# Structural and biochemical characterization of in vivo assembled *Lactococcus lactis* CRISPR-Csm complex

**DOI:** 10.1038/s42003-022-03187-1

**Published:** 2022-03-29

**Authors:** Sagar Sridhara, Jay Rai, Charlisa Whyms, Hemant Goswami, Huan He, Walter Woodside, Michael P. Terns, Hong Li

**Affiliations:** 1 Institute of Molecular Biophysics, Florida State University, Tallahassee, FL 32306 USA; 2 Department of Chemistry and Biochemistry, Florida State University, Tallahassee, FL 32306 USA; 3 Department of Microbiology, University of Georgia, Athens, GA 30602 USA; 4 Department of Biochemistry and Molecular Biology, University of Georgia, Athens, GA 30602 USA; 5 Department of Genetics, University of Georgia, Athens, GA 30602 USA; 6 Present Address: Department of Medical Biochemistry and Cell Biology, University of Gothenburg, Gothenburg, 40530 Sweden

**Keywords:** Cryoelectron microscopy, RNA, CRISPR-Cas9 genome editing

## Abstract

The small RNA-mediated immunity in bacteria depends on foreign RNA-activated and self RNA-inhibited enzymatic activities. The multi-subunit Type III-A CRISPR-Cas effector complex (Csm) exemplifies this principle and is in addition regulated by cellular metabolites such as divalent metals and ATP. Recognition of the foreign or cognate target RNA (CTR) triggers its single-stranded deoxyribonuclease (DNase) and cyclic oligoadenylate (cOA) synthesis activities. The same activities remain dormant in the presence of the self or non-cognate target RNA (NTR) that differs from CTR only in its 3′-protospacer flanking sequence (3′-PFS). Here we employ electron cryomicroscopy (cryoEM), functional assays, and comparative cross-linking to study in vivo assembled mesophilic *Lactococcus lactis* Csm (LlCsm) at the three functional states: apo, the CTR- and the NTR-bound. Unlike previously studied Csm complexes, we observed binding of 3′-PFS to Csm in absence of bound ATP and analyzed the structures of the four RNA cleavage sites. Interestingly, comparative crosslinking results indicate a tightening of the Csm3-Csm4 interface as a result of CTR but not NTR binding, reflecting a possible role of protein dynamics change during activation.

## Abstract (web summary)

This study presents Cryo-EM structures of the *Lactococcus lactis* Type III-A CRISPR-Cas effector complex in three functional states, including bound forms with cognate and non-cognate target RNA. The results suggest changing protein dynamics during effector complex activation.

## Introduction

Small RNAs play a wide range of functional roles in microbiota, from transcription termination, stress response, metabolic regulation to immunity through interactions with proteins, nucleic acids, and small molecule partners^1–3^. Among these, the emerging CRISPR (Clustered Regularly Interspaced Short Palindromic Repeats) RNA-based immunity affords the most complex processes of RNA regulation. CRISPR is a characteristic genetic feature of bacteria and archaea required to mount immunity against invading nucleic acids originating from viral infections or invasion of plasmids or other mobile genetic elements^4–7^. CRISPR RNA (crRNA) partners with CRISPR-associated (Cas) proteins to interfere against the invading nucleic acids. Currently, known CRISPR-Cas systems are remarkably broad both in composition and interference mechanisms. Understanding the molecular basis of CRISPR-Cas systems not only unravels the most fundamental RNA-mediated biochemical mechanisms underlying the microbiological warfare but also leads to novel biotechnological applications.

Among the known types of CRISPR-Cas systems, type III is unique in that it elicits multifaceted immune responses. Type III systems are triggered by actively transcribing viral messenger RNA (mRNA) that bears sequence complementarity to the crRNA carried by type III effectors^8^. Upon activation, type III systems directly degrade invader mRNA and its encoding DNA and more surprisingly, synthesize cyclic oligoadenylates (cOAs) that generate secondary antiviral responses^9,10^. The viral RNA-stimulated DNA degradation activity is recently shown to increase host mutagenesis and accelerate antibiotic resistance^11^. The molecular mechanism of Type III systems is, therefore, arguably the most complex among all types of known CRISPR-Cas systems. Other than being valuable model systems for studying CRISPR-Cas immunity, the Type III effectors have found applications in nucleic acid detection with high sensitivity^12–14^. A molecular understanding of type III systems, therefore, provides the basis for unveiling their complex mechanism as well as improving the utility in nucleic acid detection.

Csm is comprised of five proteins: Cas10, or Csm1, Csm2-Csm5, and a crRNA. The single-stranded DNase (ssDNase) and the cOA synthesis activities reside within the Csm1 subunit^9,10,15–17^ and the RNase activity resides within Csm3^17–19^. Although the Csm3-mediated RNase activity requires the mere crRNA-target RNA complementarity, the Csm1-mediated DNase and cOA synthesis are allosterically regulated^20^. The binding of a cognate target RNA (CTR) that complements the crRNA guide region but not the 8-nucleotide (nt) repeat-derived 5′-crRNA tag sequence (alternatively referred to as a 5′-handle), induces non-specific DNase and the cOA synthesis activities^15,17,18,20^. In contrast, a target RNA that complements both the guide region and the 5′-tag of the crRNA (alternatively referred to as the non-cognate target RNA, or NTR) is unable to switch on the Csm1-mediated activities^15^^,^^17–19^. In other words, NTR represents “self” RNA and thus prevents Csm-mediated autoimmunity. Interestingly, cleavage of CTR by Csm3 causes cessation of the Csm1-mediated activities, thereby providing a temporal regulation of DNase activity that avoids the potential cleavage of the host genome^15,21^. Furthermore, although isolated Csm1 is a constitutively active DNase^22,23^, the Csm assembly (apo effector) possesses no such activity, indicating that both Csm assembly, as well as target RNA, regulate the enzymatic activities of Csm1.

Recently, several structures of Csm complexes bound with their respective CTR and NTR in the presence and absence of nucleotides were solved^24–29^. In addition, high-resolution crystal structures of individual subunits or subcomplexes from a number of species with or without bound nucleotides are known^23,30,31^. These structural data and the associated biochemical analyses have created an opportunity to begin to resolve the molecular mechanism of Csm activation. The observed structural transitions from the apo to the NTR- or the CTR-bound state provide consistent explanations for the activity and regulation of target RNA cleavage, but less satisfactorily for the cOA synthesis and the DNase activities. Only subtle conformational rearrangements were observed at either the cOA synthesis or the DNase active center when comparing the CTR- and the NTR-bound structures. As a result, structural dynamics was proposed to be a method of regulation^24,25,30^. However, structural evidence entailing molecular dynamics-based regulation remains unavailable.

A recent single-molecule fluorescence resonance energy transfer (FRET) study eloquently showed that the Csm differs in its dynamics when bound with model CTR or NTR, respectively^32^. In the presence of CTR, whereas the FRET efficiency of the donor-acceptor pair anchored on the 3′ end of the target and Csm4 remained steady, that measured on the donor-acceptor pair anchored on the 3′ end of the target and Csm1 fluctuated over a large range, indicating a dynamic motion in Csm1 with respect to Csm4. In the presence of NTR, in contrast, the FRET efficiency from the same FRET pair had less fluctuation, suggesting reduced dynamics in Csm1 correlated with its inhibited state^32^. When combined with the minor rearrangements between the CTR- and NTR-bound Csm structures, the single molecular FRET data suggest that dynamics provide a biophysical basis for the foreign RNA-mediated activation of Csm1. However, given that each FRET records a single geometric parameter, the dynamic activation model must be strengthened by 3D structural analysis of the entire Csm complex.

In addition to RNA-mediated control, metabolites such as metal ions and ATP may also influence the function of Csm. In previously studied thermophilic Csm structures^24,25^, the 3′ PFS of CTR was only observed in the presence of ATP or its non-hydrolyzable analog, suggesting a stabilizing role of ATP in 3′ PFS binding to Csm1. ATP binds to the conserved Palm domain in Csm1 and is also stabilized by a hairpin loop in Csm4^24,25^. As the first few nucleotides of the 3′ PFS also bind to the Palm domain, these structural observations raised a question if ATP is required for 3′ PFS binding and thus regulates the activity of Csm. Understanding how to target RNA, ATP, metal ions, and the Csm subunits juxtapose, and the functional consequences are required to understand how Csm activities are controlled.

The mesophilic *Lactococcus lactis* Csm (LlCsm) was previously shown to function in bacteria as a Type III-A defense system^16,33^. Recently, the in vivo reconstituted LlCsm complexes from an all-in-one expression system were successfully repurposed for sensitive detection of SARS-CoV-2 virus with high sensitivity^13^. To understand the molecular mechanisms of LlCsm, we characterized its structures both in solution and by cryo-EM. We report the structures of the apo, the NTR-, and the CTR-bound LlCsm complexes that collectively represent the active and the inactive states at 2.9–4.5 Å resolutions. We observed two forms of the apo complex and captured the 3′ PFS of CTR-bound to LlCsm in absence of ATP. We also probed solution properties of LlCsm by lysine-specific cross-linking and a dual-channel fluorescence assay, together with separately purified LlCsm6, that simultaneously detects the DNase and cOA_n_ synthesis activities. Our structural and functional analysis identified differences between LlCsm and previously characterized Csm complexes in both stoichiometry and response to ATP binding.

## Results

### Target RNA and ATP regulate LlCsm activities

**Fig. 1:**
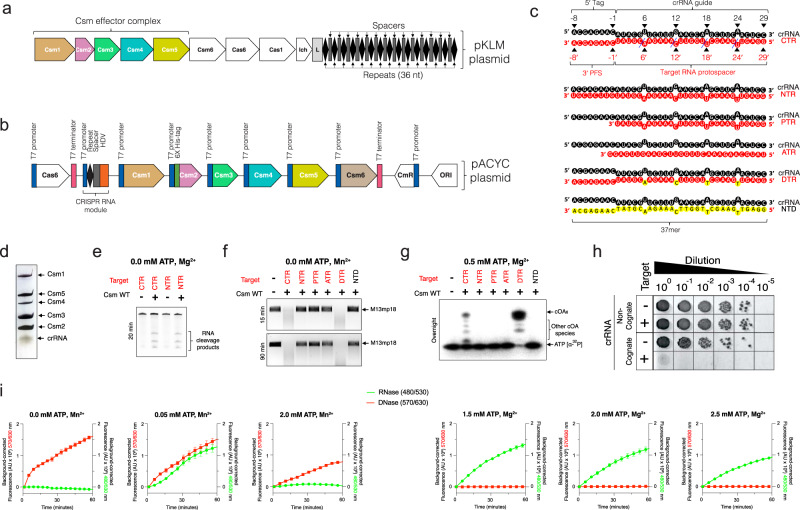
Purification and activity of *Lactococcus lactis* type III-A (Csm) CRISPR-Cas system (**a**) Genome organization of Type III-A CRISPR-Cas system in pKLM plasmid of *Lactococcus lactis*. The locus harbors tandem copies of 36 nt repeats (diamonds) and spacers (rectangles) of varying lengths. **b** Schematic representation of pACYC expression plasmid harboring *L. lactis* CRISPR-Cas genes Csm1-6, Cas6, and CRISPR RNA locus in a HDV ribozyme construct. **c** Schematic representation of crRNA and various crRNA-target RNA duplexes used in the study (*crRNA*: CRISPR RNA, *CTR*: cognate target RNA, *NTR:* non-cognate target RNA, *PTR:* protospacer target RNA, *ATR:* anti-sense target RNA, *DTR:* deoxy-substituted target RNA, *NTD:* non-target DNA). The blue dashed lines indicate target RNA cleavage sites. Deoxynucleotides are highlighted in yellow. **d** Silver stain profile of purified LlCsm RNP showing Csm1-5 proteins and crRNA. **e** In vitro RNA cleavage assay by WT LlCsm complex. The reactions were performed at 37 °C for 20 min and contained 100 nM LlCsm complex and 500 nM target RNA. **f** In vitro DNA cleavage assay by WT LlCsm complex. The reactions were carried out with 100 nM LlCsm complex, 200 nM target RNA on M13mp18 at 37 °C for 10–90 min as indicated in a cleavage buffer containing 33 mM Tris-acetate pH 7.6 (at 32 °C), 66 mM potassium acetate, and 10 mM MnCl_2_. **g** Urea-polyacrylamide gel electrophoresis (Urea-PAGE) showing cOA synthesis by LlCsm complex. The reactions were carried out at 37 °C overnight with 100 nM LlCsm complex, 200 nM target RNA, 500 μM cold ATP spiked with [α-^32^P]-ATP in a buffer containing 33 mM Tris-acetate pH 7.6 (at 32 °C), 66 mM potassium acetate, and 10 mM MgCl_2_. **h** In vivo plasmid interference assays with the wild type LlCsm harboring a non-cognate crRNA or the wild type LlCsm harboring a cognate crRNA. **i** Dual fluorescence assay using DNA and RNA oligo reporters with 5′ Alexa Fluor 594 fluorophore/3′ Iowa Black RQ quencher and 5′ 6-FAM fluorophore/3′ Iowa Black FQ quencher, respectively. The Csm1-mediated DNase and the Csm6-mediated RNase are simultaneously detected at 570/630 nm excitation/emission (red) and 480/530 nm excitation/emission (green) wavelengths, respectively. Target RNA (CTR) was used as the stimulator at 500 nM, LlCsm at 200 nM, LlCsm6 at 1 nM, and metal ions at 10 mM. Curves are averages with error bars of three technical replicates subtracted by the signals from water-stimulated reactions.

We established both in vitro and in vivo enzymatic assays to dissect the mechanism of LlCsm activation. We modified a previously described multi-protein-RNA co-expression system^16^ to produce LlCsm ribonucleoproteins (RNPs) that represent those present in *L. lactis* (Fig. 1a–c). This construct allows co-expression and concerted processing of the repeats by Cas6 endoribonuclease and of the spacer by the HDV ribozyme that gives rise to a mature 37-nt crRNA bound to the purified LlCsm complex (Fig. 1d & Figure S1). The N-terminally His-tagged Csm2 subunit enabled the isolation of the entire LlCsm complex via metal-affinity and size-exclusion chromatography. The final homogenous LlCsm complex was verified to contain Csm1-5 and the 37-nt crRNA (Fig. 1d & Figure S1) and was used in subsequent activity assays and structural studies. As previously observed^33^, Csm6 is not a stable component of the Csm complex (Fig. 1d & Figure S1).

We employed the traditional assay methods in measuring LlCsm activities using various target RNAs (Fig. 1c). Electrophoretic analysis showed that similar to what was observed for other homologous Csm complexes, the in vivo assembled LlCsm possesses CTR-induced Csm3-mediated target RNA cleavage (Fig. 1e, Figure S1), the Csm1 HD domain-mediated ssDNA cleavage (Fig. 1f, Figure S1), the Csm1 GGDD motif-mediated cOA_6_ synthesis (Fig. 1g) that was identified to be primarily cOA_6_ (Figure S1). Other non-specific target nucleic acids such as non-complementary anti-sense target RNA, protospacer-only target RNA (PTR), or cognate target DNA failed to elicit any Csm1 functionality (Fig. 1). Noteworthy, the deoxy target RNA (DTR) whose cleavage site 2’-hydroxyl groups are replaced by hydrogen, induced a greater extent of ssDNA cleavage (Fig. 1f) and cOA_6_ synthesis (Fig. 1g) consistent with previous observations that cleavage of CTR reduces ssDNA cleavage. These assays show that LlCsm cleaves both the CTR and the NTR but is only active in DNA cleavage and cOA_6_ synthesis with the CTR.

In bacterial cells, LlCsm also exhibited plasmid interference only upon expression of CTR and with intact GGDD motif in Csm1 (Fig. 1h, Table S1, Figure S2a and S2b). Interestingly, the removal of Csm2 subunit (ΔCsm2-LlCsm) impaired the interference activity regardless of if Csm1 is mutated (Figure S2c). In vitro, the ΔCsm2-LlCsm complex could be isolated in a stable form (Figure S2d) and was shown to have severe defects in target RNA cleavage (Figure S2e) and low levels of DNase and cOA_6_ synthesis activities (Figure S2f and S2g).

To assess the relationship between the two activities within the Csm1 subunit, we simultaneously detected the DNase and the cOA_6_ synthesis activities of the LlCsm-Csm6 system by a dual-channel fluorescence assay (Fig. 1i). The DNase activity is measured by a DNA oligo covalently linked to a fluorophore (5′-Alexa Fluor 594 N) and a quencher (3′ Iowa Black RQ). The cOA_6_ synthesis activity is measured through cOA_6_-stimulated cleavage of an RNA oligo linked to a fluorophore (5′ 6-FAM) and a quencher (3′ Iowa Black FQ) by LlCsm6. The fluorescence assay confirmed the DNase and cOA_6_ synthesis activities and in addition, allowed us to probe the interplay between the two independent activities under different metal ions and ATP concentrations. In presence of Mn^2+^ and a low concentration of ATP, LlCsm elicited both strong DNase and the cOA_6_-mediated RNase activities (Fig. 1i). Interestingly, both activities were reduced as ATP concentration increased (Fig. 1i). In presence of Mg^2+^ and ATP, the cOA_6_-mediated RNase was the primary activity. This activity was also reduced as ATP concentration increased (Fig. 1i), similar to what was observed under multiple reaction conditions when the LlCsm system was optimized for SARS-CoV-2 detection^13^. These results are consistent with a requirement of Mn^2+^ for the DNase and the preference for Mg^2+^ in cOA_6_ synthesis by LlCsm. Importantly, they reveal that ATP reduces both LlCsm-mediated enzymatic activities.

### Distinct LlCsm structural assemblies

To understand the molecular mechanism of LlCsm activation, we employed cryo-EM to determine the structures of the LlCsm complexes alone or bound with the NTR or CTR (Figures S3, S4, S5 and S6). The LlCsm complexes harboring Csm1 Asp14Asn and Csm3 Asp30Ala mutations were used in cryo-EM studies in order to prevent degradation of the bound nucleic acids. The apo, the NTR-bound, and the CTR-bound complexes were prepared similarly prior to making cryo-grids. The co-purified LlCsm-crRNA complex was pre-incubated with or without CTR or NTR in molar excess (RNP:Target RNA = 1:2) followed by fractionation on a size-exclusion column (Figure S1). The peak fractions of each run containing the complex were suitably diluted before plunging to achieve homogenous particle distribution. Data collection and the single-particle reconstruction resulted in five main structures: two apo structures with different stoichiometry (4.5 Å and 2.9 Å overall), the NTR-bound structure (3.4 Å overall), and the two CTR-bound structures with different subunit stoichiometry (3.0 Å and 3.3 Å overall) (Methods).

The electron potential densities for Csm3, Csm4, crRNA, and the target RNA, if bound, have excellent quality in all structures to allow tracing and placement (Figure S6). The full-length Csm1 could be reliably traced in both the high-resolution apo or the NTR structure while focused refinement with a Csm1 mask in the CTR structure allowed its placement (Figure S6). A large majority of Csm5 was also traced in all three structures. All final structures except for the 4.5 Å apo structure was refined to satisfactory correlation coefficients and geometry (Table S2). The 4.5 Å apo structure was only used to confirm the presence and placements of all subunits that were traced elsewhere.

**Fig. 2:**
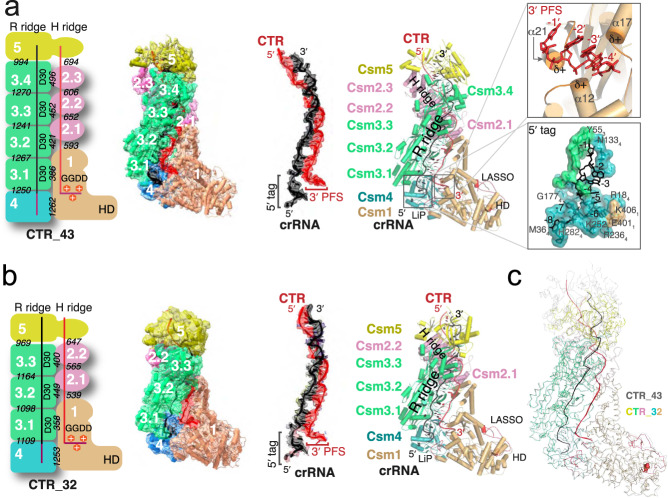
Structures of Cognate target RNA (CTR)-bound LlCsm complex. Numbers in white indicate names of the subunit where N or N.m represent CsmN (or the mth CsmN). Key secondary elements are labeled. The crRNA and CTR are colored black and red, respectively. **a** Schematic representation, electron potential density, and cartoon representation of CTR-43 with stoichiometry 1_1_2_3_3_4_4_1_5_1_crRNA_1_Target_1_. Italicized numbers in black indicate the buried solvent-accessible surface of the corresponding subunits in Å^2^. The relative positions of crRNA 5′-tag and the CTR 3′-PFS are highlighted. The boxed image at the top displays three α-helices of LLCsm1: α12, α17, and α21 that orient the positively charged N-terminus towards the 3′-PFS of CTR, thus forming a favorable protein-RNA interaction. The boxed image towards the bottom displays the Csm3-Csm4 pocket where the 5′-tag of crRNA traverses. **b** Schematic representation and overall structure of CTR-32 with stoichiometry 1_1_2_2_3_3_4_1_5_1_R_1_T_1_. The crRNA and CTR are colored black and red, respectively. The relative positions of crRNA 5′-tag and the CTR 3′-PFS are highlighted. **c** Ribbon representation of CTR-43 (gray) and CTR-32 (colored) with their Csm3 subunits superimposed highlighting structural differences in other subunits.

Two distinct CTR-bound structures were resolved from two major classes that have 1_1_2_3_3_4_4_1_5_1_crRNA_1_Target_1_ (CTR-43) and 1_1_2_2_3_3_4_1_5_1_crRNA_1_Target_1_ (CTR-32) stoichiometry, respectively, where each number corresponds to a numbered Csm protein (Fig. 2, Figure S3). Interestingly, unlike previously determined thermophilic Csm structures that prefer assemblies with fewer numbers of Csm3 and Csm2^24,25^, LlCsm prefers the longer CTR-43. Like other Csm, LlCsm is essentially comprised of two helical ridges capped at the foot by the characteristic subunit Csm1. One ridge is made of RAMP (Repeat Associated Mysterious Repeat) protein or domains (R ridge) and the other is made of helical proteins (H ridge). Four Csm3 subunits, the RAMP domain of Csm4 (at the foot) and that of Csm5 (at the head) comprise the R ridge whereas three Csm2 subunits, the helical domain (domain D4) of Csm1 (at the foot) and that of Csm5 (at the head) comprise the H ridge (Fig. 2). The crRNA traverses along and buries a large interface with the R ridge (Fig. 2). The CTR, in contrast, traverses along and buries a relatively small interface with the H ridge (Fig. 2). The smallest buried surface area is found between pairs of Csm3 and Csm2 subunits (~420 Å). As interaction strength is correlated with the amount of buried solvent-accessible area^34^, the R ridge and its interaction with crRNA is thus more stable than the H ridge and its interaction with the target RNA.

**Fig. 3:**
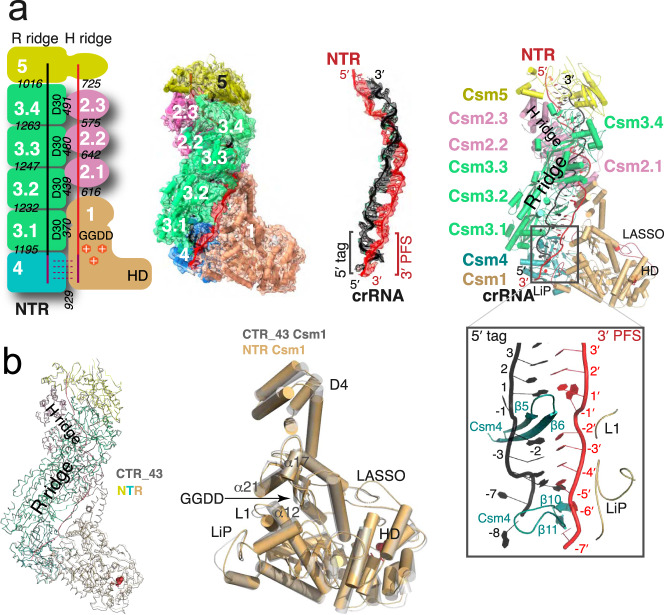
Structure of the non-cognate target RNA (NTR)-bound LlCsm complex. Numbers in white indicate names of the subunit where N or N.m represent CsmN (or the mth CsmN). Key secondary elements are labeled. The crRNA and CTR are colored black and red, respectively. **a** Schematic representation and electron potential density of NTR complex with stoichiometry 1_1_2_3_3_4_4_1_5_1_crRNA_1_Target_1_. Italicized numbers in black indicate the buried solvent-accessible surface of the corresponding subunits in Å^2^. The relative locations of crRNA 5′-tag and the NTR 3′-PFS are highlighted. The boxed image displays the base-paired 5′-tag and 3′-PFS surrounded by Csm4 and Csm1 structural elements. **b** Comparison of CTR-43 and NTR complex in ribbon representations. The Csm3 subunits of the two complexes are superimposed to reveal, if any, differences in other subunits. Csm1 subunits from both complexes are compared in a cartoon representation. Note the different locations of the LiP loop in the two structures.

**Fig. 4:**
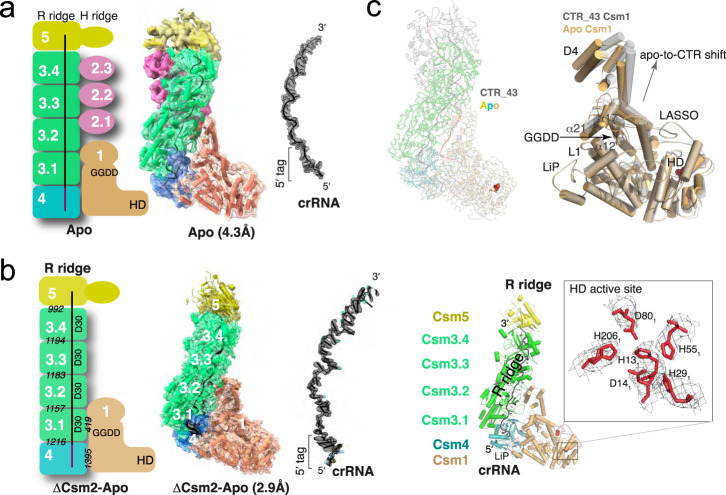
Structures of apo LlCsm complexes lacking a bound target RNA. Numbers in white indicate names of the subunit where N or N.m represent CsmN (or the mth CsmN). Key secondary elements are labeled. The crRNA is shown in black. **a** Schematic representation, electron potential density, and cartoon representation of the LlCsm apo complex with stoichiometry 1_1_2_3_3_4_4_1_5_1_crRNA_1_Target_0_ determined as a subpopulation when incubated with DTR. **b** Schematic representation, electron potential density, and cartoon representation of the refined LlCsm apo complex with stoichiometry 1_1_2_0_3_4_4_1_5_1_crRNA_1_Target_0_ determined when incubated with no target RNA. Italicized numbers in black indicate the buried solvent-accessible surface of the corresponding subunits in Å^2^. The boxed image shows a close-up view of well-placed catalytic HD residues with electron potential density. The putative catalytic residues His13, Asp14, Asp80, and His206 form a permuted HD motif and are strongly conserved. **c** Ribbon representation of apo LlCsm complex (at 2.9 Å and colored) overlaid with LlCsm CTR-43 complex (gray) using their Csm3 subunits. The HD residues are highlighted in red. The cartoon representation depicts an upward shift of Csm1 D4 domain upon binding to CTR wherein alpha-helices α12, α17, and α21 follow along. Note the minimal shifts among LASSO, LiP, and L1 loops.

The same stoichiometry is also observed in the NTR complex (Fig. 3 and Figure S4). However, unlike the CTR complex, the Csm1 subunit is well ordered in NTR and allowed its clear tracing. Interestingly, we obtained two apo structures with different stoichiometry. The sample made from a mixture of LlCsm with DTR only resulted in a homogenous subpopulation without DTR bound. This apo form of LlCsm was refined to 4.5 Å resolution and contains the same number of protein subunits as in the CTR- and the NTR-bound complexes (Fig. 4). A second apo structure was also obtained from LlCsm without incubating with a target RNA that was refined to 2.9 Å and contains all but the Csm2 subunits (1_1_2_0_3_4_4_1_5_1_crRNA_1_Target_0_) (Fig. 4, Figure S5). As all Csm subunits were present immediately before making the cryoEM grids (Figure S1), the reason for the lack of Csm2 in the 2.9 Å apo complex is likely due to its dissociation from the complex during plunge freezing. Nearly all particles that went into the reconstruction of the apo structure contain no Csm2 (Figure S5). Notably, both the CTR-bound and the NTR-bound particles contain a large subpopulation without a target RNA that also lacks the LlCsm2 subunits (Figures S3 and S4). Though still not clear what conditions led to the capture of the apo LlCsm in two different stoichiometries, it indicates instability of Csm2 associated with the apo complex. In summary, the five distinct structures of LlCsm among its different functional states reflect a differential binding strength that depends on the presence or the absence of self or foreign target RNA.

### RNase centers link LlCsm2 to LlCsm1

**Fig. 5:**
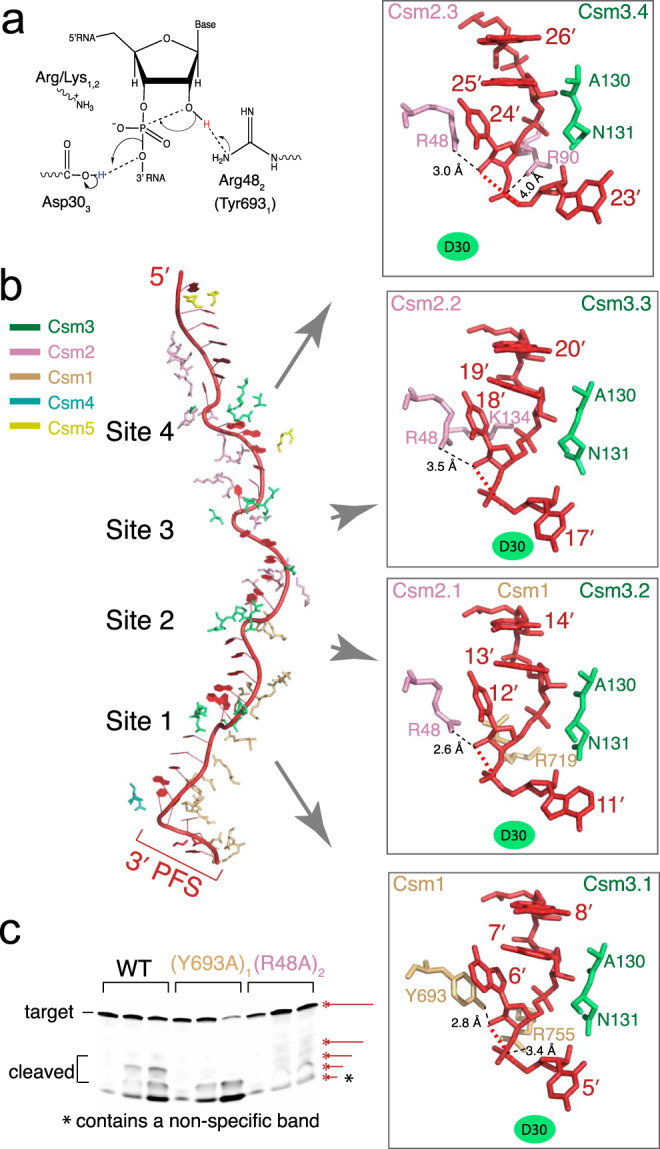
Structures of the four target RNA cleavage centers. **a** A possible reaction mechanism mediated by the conserved Csm3 (Asp30) and Csm2 (Arg48) residues. **b** Each cleavage center is superimposed and displayed in close-up view at the same orientation in the inset. The residues of Csm1, Csm2, and Csm3 surrounding the cleavage sites are shown in stick models using the same color scheme as in structural models and labeled. The mutated Asp30Ala (Csm3) is shown as a filled oval. Upon binding to the crRNA, target RNA undergoes base flipping every 6th nucleotide at the four cleavage sites (sites 1–4), forming an inline geometry involving the 2’-nucleophile oxygen, the scissile phosphate, and the 5′-leaving oxygen atoms. **c** In vitro cleavage results with 5′ Cy3-labeled target RNA. Possible products are indicated by starred red lines. A non-specific product is indicated by a star.

Previous kinetic analysis showed that both the cOA_6_ synthesis and DNase activities dampen with the progression of target RNA cleavage^15,21^. Further, we showed that a non-cleavable target RNA yielded stronger cOA_6_ and DNase activities than a cleavable target RNA did (Fig. 1f, g, Figure S2). To shed light on the mechanistic link between RNA cleavage and Csm1 activities, we analyzed the structure of the RNase centers of LlCsm. Although Csm requires a divalent ion to cleave its target RNA^15,18^, the structural arrangement of the observed target RNA cleavage centers and the fact that the cleavage products of the analogous Type III-B (Cmr complex) has 2′,3′-cyclic phosphate termini^35^ suggest that it follows an RNase A-like cleavage mechanism^36^ similar to that of the crRNA processing endoribonuclease Cas6^37^. This reaction mechanism is also consistent with our observed increase in LlCsm activities if the 2’-hydroxyl group upstream of scissile phosphate is replaced by hydrogen (Fig. 1f, g). Four RNase centers are observed in the CTR-43 complex along the backbone of the bound CTR (Fig. 5). The RNase sites are marked by the thumb loop of the Csm3 subunits that harbors the critical Asp30 residue. However, as the Asp30Ala mutant was used to form the cryoEM sample, Asp30 was not observed and the substituted Ala30 is 4–5 Å away from the leaving group oxygen. Despite so, the thumb loop-facilitated flipping of the nucleotide, 5′ of the cleavage sites enable a favorable conformation for cleavage. The 2′-nucleophilic oxygen, the scissile phosphate group, and the leaving group oxygen form the classic inline geometry at each of the four sites (Fig. 5). Either an arginine (sites 2–4) or a tyrosine (site 1) residue is situated near the 2′-nucleophilic oxygen that could act as the general base to extract the proton. Arg48 of Csm2 is on α2 and surrounded largely by non-polar residues including Leu51 of Csm2, Ile26, Gly27, and Ala28 of Csm3, which may help to reduce its pKa. Studies of a number of other enzymes including Cas6 have also provided evidence supporting arginine’s role as a general base^38,39^. Consistently, mutation of Tyr693 to alanine (Y693A) in Csm1 resulted in impaired cleavage at site 1 while that of Arg48 to alanine in Csm2 (R48A) seemed to have abolished cleavage at the other three sites (Fig. 5). Mutation of corresponding residues in previously studied *Streptococcus thermophilus* Csm (Figure S7) was also found to diminish or reduce the cleavage activity^25^. A positively charged residue (lysine or arginine) is observed to be placed near the scissile phosphate that could stabilize the negative charge developed on the penta-coordinated phosphate. Consistently, reduced target RNA cleavage was reported in the equivalent MjCsm2 Lys127Ala and AfCmr5 Lys148Ala mutants (Figure S8)^40^. However, mutation of the equivalent arginine residues in StCsm1 placed near sites 1 and 2 (Figure S7) did not impact RNA cleavage^24,25^. Alternatively, the required divalent metal ions could act to stabilize the developing negative charge at the transition state. Asp30, if it were present, could act as the general acid by donating a proton to the leaving oxygen. Asp30 may also participate in the coordination of a divalent metal that enables a water molecule to donate the proton. Mutation of the strongly conserved Asp30-equivalent residue in several Csm3 homologs was also found to be deleterious to RNA cleavage (Figure S8). The putative general base arginine or tyrosine supplied by Csm2 (sites 2–4) and Csm1 (site 1), respectively, suggests a critical role of Csm2, in addition to Csm3, towards achieving target RNA cleavage.

### The structure of 3′-PFS in absence of ATP

Interestingly, previous structural studies of Csm complexes did not capture 3′ PFS of CTR without the aid of ATP or its analog binding^24,25^ (Figure S9). In contrast, though no ATP was used in formation of CTR-bound LlCsm, we observed 3′-PFS traversing through Csm1 (Fig. 2a and Figure S9). Four nucleotides, C(-1)′-A(-2)′-A(-3)′-G(-4)′ of the 3′ PFS in CTR make a 90 degree turn from the trajectory of the rest of the target RNA and rests on Csm1 (Fig. 2a & Figure S9). When the CRISPR RNA of the LlCsm was superimposed with that of the StCsm-ATP complex (6IG0), a slight difference in the 3′ PFS trajectory between the two complexes was observed. The LlCsm 3′ PFS tracks closer while StCsm 3′-PFS is further away from the body of Csm1 (Figure S9). Notably, three helices: α12 of the Palm 1 domain, α17 and α21 of the Palm 2 domain of LlCsm1 (Fig. 2a & Figure S9) point their N-terminal ends towards the phosphate backbone of the 3′ PFS nucleotides, thereby forming a positive groove that interacts favorably with the negatively charged 3′-PFS phosphate backbone in a sequence-independent manner. The 3′-PFS-helix dipole interaction locks the two Palm domains in preparation for Csm1-mediated activities. Consistently, the target RNA lacking 3′ PFS (PTR) failed to induce either cOA_6_ synthesis or DNase activity of LlCsm1 (Fig. 1f, g).

The 3′-PFS of NTR differs from that of CTR-43 and bears a complementary sequence to that of the crRNA 5′ tag. Seven of the eight PFS nucleotides are observed and only four form base pairs with the 5′ tag (Fig. 3). To facilitate further comparison of key structural elements, we assigned common names to three Csm1 loops: the LASSO loop (LlCsm1 residues 86–103), the L1 loop (LlCsm1 residues 263–269), the Linker-Palm1 (LiP) loop (LlCsm1 residues 394–416) and one Csm4 loop: the Lid loop (LlCsm4 residues 82–96) (Figure S7). Nucleotide (-1)′ is flipped out by the thumb loop of Csm4 similar to those at the cleavage sites. Nucleotides (-2)′–(-5)′ base pair with nucleotides (2–5) of the 5′ tag and (-6)′–(-7)′ rest on a groove formed by the Lid loop of Csm4 and the LiP loop of LlCsm1 (Fig. 3). Though the Csm1-Csm4 interface further reduces from 1261 Å^2^ in the CTR-bound to 929 Å^2^ in the NTR-bound complex, LlCsm1 gains 433 Å^2^ interfaces with 3′-PFS that in turn bases pairs with the 5′ tag of crRNA (Fig. 3b). As a result, 3′-PFS establishes an extensive interface that stabilizes Csm1 onto Csm4 and crRNA.

### Structural changes among different LlCsm complexes

Given the close resemblance in Csm1 structure between the CTR- and the NTR-bound LlCsm and the large distance between the 3′-PFS binding site and the HD domain (Figs. 3 and 4), the strikingly different enzymatic activities between the two may be accounted for by their different dynamic behaviors. The refined atomic displacement parameter, or the B factor, combined with the map-fitting quality score, Q score^41^, provides an indication that the Csm1 subunit is less stable in the CTR- than in the NTR-bound complex (Figure S10). Accompanying the increase in the B factor for Csm1 is a decrease in B factors of the Csm2-based H ridge and vice versa (Figure S10). Thus, when Csm1 is well ordered such as in the apo state, Csm2 is loosely associated with the complex, which could lead to its disassociation (Fig. 4). Furthermore, a comparison of 2D classes of the apo, CTR-43, and NTR particles provides some direct evidence of the structural difference of the HD domain. A large number of particles of the CTR-43 but not those of the other two complexes lacked clearly defined side views of the HD domain (Figure S10c), which could indicate a change in the structural property of the HD domain when CTR is bound.

**Fig. 6:**
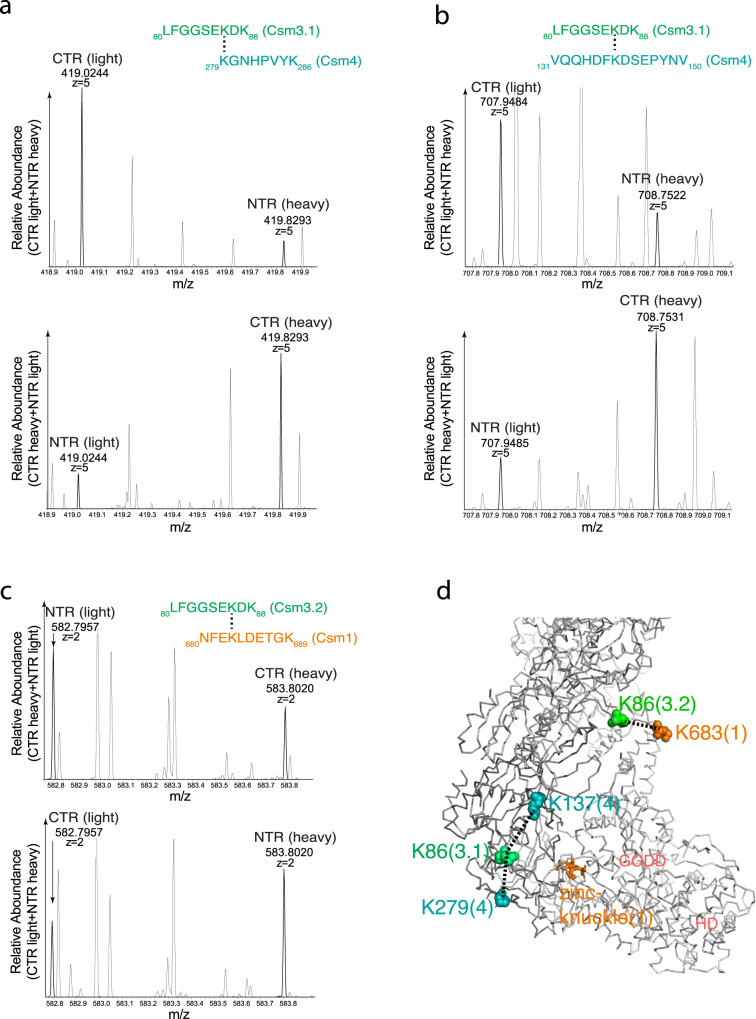
Comparative cross-linking on LlCsm CTR and NTR complexes. Mass spectra of trypsin-digested and BS3 (bis(sulfosuccinimidyl) 2,2,7,7-suberate)-cross-linked peptides of the CTR-bound and NTR-bound LlCsm that exhibited a significant difference in abundance. The deuterated BS3-d4 is designated as heavy and the non-deuterated BS3-d0 is designated as light, respectively. The mass and the ion state are indicated to the known accuracy of the instrument. **a** The mass spectra and the peptide sequences match the identified crosslinks (darker peaks) for the cross-linked Csm3.1 Lys86 and Csm4 Lys279 (3.1 denotes the Csm3 subunit closest to Csm4). **b** The mass spectra and the peptide sequences match the identified crosslinks (darker peaks) for the cross-linked Csm3.1 Lys86 and Csm4 Lys137. **c** The mass spectra and the peptide sequences match the identified crosslinks (darker peaks) for the cross-linked Csm3.2 Lys86 and Csm1 Lys683. **d** Mapping of the cross-linked lysine pairs shown in panels A-C onto the CTR-43 structure. Dashed lined indicate the residue-residue distances around 11.4 Å. Key regions in Csm1 are labeled.

To probe structural differences of LlCsm at the three different functional complexes in solution, we performed lysine-specific cross-linking and compared the abundance of cross-linked pairs throughout the entire LlCsm complex between CTR- and NTR-bound. We took advantage of isotopically labeled crosslinker bis(sulfosuccinimidyl)suberate (BS3) (linker arm length 11.4 Å) in accurately identifying the cross-linked lysine pairs and their relative abundance in presence of bound CTR or NTR (Methods). We identified a total of 24 pairs of cross-linked lysine residues either between or within subunits for both the CTR- and NTR-bound complexes (Table S5). Among these, three pairs of lysine residues, Lys86 (Csm3.1)-Lys279(Csm4), Lys86 (Csm3.1)-Lys137(Csm4), and Lys86(Csm3.2)-Lys683(Csm1) exhibited significant abundance difference between the CTR- and the NTR-bound complexes (Fig. 6, Table S5, Figure S11). The Lys86(Csm3.2)-Lys683(Csm1) is enhanced in the NTR-bound complex while the Lys86 (Csm3.1)-Lys279(Csm4), Lys86 (Csm3.1)-Lys137(Csm4) pairs are enhanced in the CTR-bound complex. Mapping these three lysis pairs on the LlCsm structure showed that they occur at the interface between Csm3.1 and Csm4 and Csm3.2 and the D4 domain of Csm1 (Fig. 6). Thus, this result suggests that CTR binding may enhance the Csm3.1-Csm4 but somewhat reduces the Csm3.2-Csm1 interface in comparison with NTR binding, consistent with the increased buried interfacial area between Csm3.1 and Csm4 in CTR-43 (Figs. 2 and 3).

## Discussion

The *Lactococcus lactis* Gram-positive bacterium is among the most extensively used microorganisms in the dairy industry and possesses a range of medical and biotechnological applications^42,43^. Although *L. lactis* was thought to rely only on various non-CRISPR-mediated defense mechanisms^44^, a novel conjugative plasmid-encoded Type III-A CRISPR-Cas system (Fig. 1a) was identified^45^. The plasmid-encoded *L. lactis* Csm (LlCsm) system was subsequently shown to confer a similar transcription-dependent plasmid DNA interference activity to those by its orthologs from *S. thermophilus*, *S. epidermidis,* and *T. thermophilus*^16,46^. While a plethora of structural information of Csm complexes has been recently made available, they represent thermophilic bacteria and archaea that are strongly enriched with the Type III systems^7^. Our study of LlCsm complex thus provides insights on the Type III-A system from a mesophilic organism previously unknown. The stable LlCsm complex prefers an assembly with longer helical ridges and seems to bind the activating 3′ PFS without the aid of bound nucleotides.

The enzymatic activities of Csm1 depend on its assembly status. In either isolated or the CTR-bound state, Csm1 is active while in the apo or the NTR-bound state, Csm1 is inactive in cleaving non-specific DNA. Exhaustive structural comparison of StCsm^25^, ToCsm^24^, and now LlCsm (this study), at different states reveal no common molecular transitions among the four states that can explain Csm1 activation. The large sliding of the Csm2-formed H ridge upon target RNA binding is shared between both the inactive NTR-bound and the active CTR-bound states. Local rearrangements within LiP or LASSO from the NTR- and the CTR-bound transition have different structural characteristics among the three Csm complexes^24,25^. Notably, despite being distant from the active sites and unconserved, mutations of LiP, L1, or LASSO do impact Csm1 activities^24,25^, suggesting that they contribute to changes of a physicochemical property of Csm1 such as protein dynamics critical to its enzymatic activities. The first experimental evidence supporting a dynamics-based activation model was presented in a single-molecule fluorescence microscopy study^32^. It showed that CTR triggers fast conformational fluctuations within Csm1 while NTR locks it in a rigid conformation^32^. Based on the diverse modes of assembly, solvent-accessible interface analysis, atomic displacement factor differences, and comparative protein cross-linking, we also observed a general trend of stability change in Csm1, and interestingly, an opposing change in Csm2 subunits upon binding different target RNA. In addition, our cross-linking results showed a change in the Csm3-Csm4 interface that accompanies target RNA binding.

We established a dual-channel fluorescence assay that is capable of monitoring both the DNase and the cOA_6_ synthesis (through LlCsm6 RNase) activities. Our results clearly show that Mn^2+^ supports both activities while Mg^2+^ mostly supports cOA_6_ synthesis (note that LlCsm6 does not require any metal ions). Interestingly, we identified an inhibitory effect of ATP on both activities under either Mn^2+^- or Mg^2+^-containing conditions. Combined with the observations that ATP stabilizes binding of 3′ PFS binding to Csm1 in two previously studied Csm complexes^24,25^, this result is consistent with the idea that elevated ATP concentrations reduce Csm activities by restricting motions of Csm1. The interference activities of the Csm system result in increased NTPs and dNTPs that may thus be used by viruses to inhibit host immunity as a negative feedback regulation.

The observed structural roles of Csm2 in target RNA cleavage and in offsetting the dynamics of Csm1 are reflected in its essentiality towards achieving in vivo immunity. Deletion of the Csm2 subunit from the effector complex was found to be detrimental to the in vivo plasmid interference activity. We noted that there is an accompanying loss of Csm5 when Csm2 is deleted. The observed disruption in immunity without Csm2, therefore, may also be contributed by the loss of Csm5 due to the previously demonstrated role of Csm5 in immunity^47^. Previously, Csm6-mediated RNase activity was implicated to be more relevant to achieving anti-plasmid immunity than Csm1-mediated DNase activity^33^. Consistently, we observed complete shutdown of plasmid interference upon mutating the Csm1 GGDD motif to GGAA (Figure S2), suggesting that cOA_6_ synthesis and consequently, Csm6 activation, is required to achieve anti-plasmid immunity in this system. The observed disruption of in vivo interference activity in absence of Csm2 suggests its role in efficient cOA_6_ synthesis.

The protein dynamics-mediated activation model also explains why Csm1 alone^22^ is active in DNase cleavage. In this case, the HD domain is not restrained and thus active. In contrast, when Csm1 is bound within the apo-Csm RNP, it is immobilized in an inactivated state. Further testing of this activation model awaits additional computational and other biophysical studies.

## Methods

### Protein expression and purification

The pACYC *Lactococcus lactis* module plasmid encoding proteins Cas6, Csm1-6, and CRISPR locus was as described previously (Fig. 1b, Tables S3 and S4)^16^. The crRNA locus in the plasmid was suitably modified to contain repeat-spacer-HDV ribozyme sequence (Fig. 1), so that Cas6 and HDV ribozyme would perform 5′- and 3′-crRNA processing, respectively. The *csm2* gene encoded an N-terminal His_6_-tag in all variants of LlCsm. The ΔCsm2-LlCsm was produced by Gibson assembly such that csm3 gene encoded an N-terminal His_6_-tag. The mutations were introduced by Q5 mutagenesis (New England Biolabs) and verified using sequencing primers (Eurofins Genomics). All variants of LlCsm ribonucleoprotein complex were produced in *Escherichia coli* NICO strain using 0.3 mM isopropyl β-d-1-thiogalactopyranoside (IPTG) for induction of protein expression. The RNP was purified using nickel-affinity chromatography followed by size-exclusion chromatography in 20 mM 4-(2-hydroxyethyl)-1-piperazineethanesulfonic acid (HEPES) pH 7.5, 200 mM NaCl, 5 mM MgCl_2_, 14 mM 2-mercaptoethanol. The fractions of the final gel filtration step containing all five Csm proteins associated with crRNA were pooled, concentrated, aliquoted, and flash-frozen using liquid nitrogen before storage in −80 °C (Fig. 1c, Figure S1b).

The His_6_-tagged LlCsm6 was separately produced for activity assays in *E. coli*. LlCsm6 was purified using nickel-affinity chromatography followed by size-exclusion chromatography in the same buffer used for LlCsm. The fractions containing homogenous LlCsm6 from the final gel filtration step were pooled, concentrated, aliquoted, and flash-frozen using liquid nitrogen before storage at −80 °C.

### In vitro assembly of RNP-target RNA complex

The 45mer NTR/CTR target RNA (Figure S1a, Table S2) with 29 bases crRNA complementarity was ordered from Integrated DNA Technologies (IDT). The purified LlCsm (D14N)_1_(D30A)_3_ effector complex was mixed with NTR/CTR at 1:2 molar ratio, incubated at 37 °C for 60 min, and resolved using an analytical Superdex S200 column (GE Healthcare) in a buffer containing 20 mM HEPES pH 7.5, 200 mM NaCl, 5 mM MgCl_2_, 14 mM 2-mercaptoethanol (Figure S1c). The peak fractions containing target-bound LlCsm complex (Figure S1d) were used to prepare cryo-EM grids. The preparation of apo-Csm complex followed the same buffer and pre-incubation conditions with the exception of no target RNA was added (Figure S1d).

### Cryo-EM sample preparation

The LlCsm RNP and RNP-target RNA samples at ~0.5 mg/ml were separately applied in 4 μL volume onto glow-discharged UltrAuFoil 300 mesh R1.2/1.3 grids (Quantifoil), blotted for 3 s at 88% humidity and flash-frozen in liquid ethane using FEI Vitrobot Mark IV. The grids were stored in liquid nitrogen before being used for imaging.

### EM data collection, processing, and 3D reconstruction

The ice-embedded LlCsm complex samples were collected on FEI Titan Krios electron microscope equipped with Gatan Bioquantum K3 direct electron detector (ThermoFisher Scientific) with the Leginon software for automatic data acquisition^48^ in a counting mode. Both motion correction and contrast transfer function (CTF) estimation were performed in RELION-3.0^49^ provide the UCSF MotionCor2^50^ and Gctf wrapper^51^. Particles were auto-picked using the LoG-based auto-picking algorithm implemented in RELION-3^49^. The stack was created and imported into cryoSPARC^52^ for 2D classification in order to eliminate bad particles. The resolution was estimated using the gold-standard Fourier Shell Correlation plot at the value of 0.143. Local resolution was estimated using Resmap^53^. All images were collected at ×81,000 magnification with 1.074 Å/pixel sampling rate at the specimen level and −1.3 to −2.8 microns defocus. A 60.07 e^−^/Å^2^–61.51 e^−^/Å^2^ dose was applied over 70–74 frames at a total exposure time of 3.09–4.5 s.

A total of 5179 images were collected from the CTR-bound complex in a movie mode (Figure S3). Images showing bad ice, astigmatism, drift, and poor sample quality were rejected resulting in 2543 images for further processing and particle picking, which resulted in a total of 2,157,655 particles. Several rounds of 2D classification led to 613,110 particles with good quality. RELION-3^49^ was used to classify the particles, which led to further reduction of particles to 229,062 based on high-resolution features. The classes with similar features were combined and auto-refined using a custom 3D mask that resulted in an overall resolution of 3.57 Å. Multiple rounds of ctf refinement were performed on RELION-3.1^54^, which improved resolution to 3.19 Å. To further resolve heterogeneity near the top of the particles, classification without alignment was performed using a mask around the Csm5 subunit, resulting in three major classes. The largest class shows no bound target RNA nor Csm2 subunit densities and therefore, no further refinement was carried out. Two other classes show strong densities of the bound target RNA and are refined to two structures with different stoichiometry (54,783 particles, CTR-43 and 62,413 particles, CTR-32). The CTR-43 class and CTR-32 class were independently further refined in RELION-3.1^54^ to a resolution of 3.27 Å and 3.35 Å, respectively. The resolution of class CTR-43 was improved to 3.07 Å with further auto-refinement with cisTEM^55^.

A total of 4382 images were collected from the apo complex in a movie mode (Figure S4) that resulted in 2336 images after the rejection of bad images. A total of 3,636,087 particles were auto picked and 2D classification was performed to remove the bad particles, which led to 1,290,536 particles. Classification with RELION-3^49^ further reduced the particles to 436,641 based on high-resolution features. The classes with similar features were combined and refined by iterative auto-refinement using a custom 3D mask and followed by multiple rounds of CTF refinement that resulted in an overall resolution of 2.98 Å. Refinement with cisTEM^55^ of the same stack improved the resolution to 2.97 Å.

A total of 3922 images were collected from the NTR-bound complex in a movie mode (Figure S5) that resulted in 1892 after the rejection of bad images. A total of 2,865,726 particles were auto-picked and 2D classified to remove the bad particles, which led to 1,036,382 particles. 3D Classification with cryoSPARC^52^ further reduced the particles to 282,878 based on high-resolution features. The classes with similar features were combined for further classification that separated the classes with and without target RNA. The classes with target RNA were combined and refined by iterative auto-refinement in RELION-3.0^49^, resulting in an overall resolution of 3.57 Å. Furthermore, like CTR refinement, classification without alignment was performed using a mask around the Csm5 subunit, to solve the heterogeneity resulting in three major classes. Two classes show no density of target RNA, and therefore no further refinement was carried out. The remaining class (39,220 particles) shows the target RNA density with four copies of Csm3 and three copies of Csm2. This class was further refined multiple rounds with CTF refinement in RELION-3.1^54^ that resulted in 3.48 Å resolution.

### Model building and refinement

The highest resolution map of the apo complex was used to build models for Csm1, Csm3, Csm4, and Csm5, and the crRNA starting from their homology-build models and manually adjusted in COOT^56^. The map of the CTR_43 complex was used to build Csm2 and the CTR and that of the NTR_43 was used to build NTR. No model was built for the 4.5 Å apo complex. Real-space refinement was carried out for all models with PHENIX^57^. For all complexes, the RELION-3 maps were first used in model building and refinement. During the early stage of the refinement, B factors for all atoms were set arbitrarily to 20.0 Å^2^. Iterative rounds of real-space refinement and manual building in COOT led to models with overall correlation coefficients to be >0.65. In the final stages of the refinement, the atomic displacement parameters (B factors) were refined with iterative rounds of positional refinement, leading to models with overall correlation coefficients to be greater than 0.8 with excellent stereochemistry parameters. These models were used to compute Q-scores with the MapQ plugin^41^ in UCSF Chimera^58^. The final models were refined against the highest resolution electron potential density either with cisTEM or RELION-3.1 (Table S2).

### In vitro target RNA cleavage assays

The RNA cleavage assays were performed in a cleavage buffer containing 33 mM Tris-acetate pH 7.6 (at 32 °C), 66 mM potassium acetate, 10 mM MnCl_2_. The reactions were performed at 37 °C for 20–150 min or as indicated and contained 100 nM LlCsm complex or 1 μM ΔCsm2-LlCsm complex and target RNA at 500 nM concentration or that linked to 5′ Cy3 fluorophore at 100 nM concentration (Table S3). The reactions were quenched using 2× formamide dye (95% formamide, 0.025% SDS, 0.025% bromophenol blue, 0.025% xylene cyanol FF, 0.5 mM EDTA). The reaction products were heated at 70 °C for 3 min and separated by 7 M Urea, 15% polyacrylamide gel electrophoresis (PAGE) gels in 1× Tris Borate EDTA (TBE) running buffer and were visualized by staining with SYBR Gold II (Invitrogen) stain or fluorescence imager.

### In vitro DNA cleavage assays

The DNA cleavage assays were performed as previously described^47^. In brief, 2 nM M13mp18 circular ssDNA (New England Biolabs) (Table S3) was treated with 200 nM LlCsm complex, 200 nM target RNA at 37 °C for 10–90 min as indicated in a cleavage buffer containing 33 mM Tris-acetate pH 7.6 (at 32 °C), 66 mM potassium acetate, 10 mM MnCl_2_. The reactions were quenched using 1× purple gel loading dye (New England Biolabs). The reaction products were heated at 95 °C for 5 min and separated on 1% agarose gel in Tris Acetic acid EDTA running buffer and were visualized by staining with ethidium bromide.

### In vitro cOA synthesis assays

The cOA synthesis assays were performed as previously described^10^. In brief, a mixture containing 160 μCi [α-^32^P]-ATP (PerkinElmer) and 500 μM ATP was incubated with 100 nM LlCsm complex, 200 nM target RNA at 37 °C overnight in a cOA synthesis buffer containing 33 mM Tris-acetate pH 7.6 (at 32 °C), 66 mM potassium acetate, 10 mM MgCl_2_. The reaction products were heat-denatured at 95 °C for 10 min and centrifuged at high speed. The supernatant was mixed with formamide dye, resolved by 8 M Urea, 24% PAGE gels in 1× TBE running buffer at 80 V for 240 min. The gels were carefully sandwiched between non-porous cellophane sheet and a porous gel drying sheet and dried for 120 min, developed for 30 min using Phosphor Screen (GE Healthcare Life Science), and visualized using the Typhoon Gel Imaging System (GE Healthcare Life Science).

To determine which cOAs are produced by LlCsm by mass spectrometry, the same reaction was carried out without [α-^32^P]-ATP. The reaction products were heat-denatured at 95 °C/10 min and centrifuged at high speed. The supernatant was analyzed by mass spectrometry on an Agilent 6230 TOF-MS with the Agilent Mass Hunter Workstation Software TOF 6500 series in positive ion mode. Spectrum was analyzed using Agilent Mass Hunter Qualitative Analysis Navigator v.B.08 and visualized using GraphPad Prism.

### Fluorescence reporter assays

The dual-channel fluorescence reporter assay was performed using a DNA probe (IDT) covalently linked to 5′-Alexa Fluor 594 (NHS Ester) fluorescent dye and 3′-Iowa Black RQ quencher and an RNA probe (IDT) covalently linked to 5′ 6-FAM (Fluorescein) fluorescent dye and 3′ Iowa Black FQ quencher (Table S3). The reactions were initiated by the addition of 500 nM target RNA to a mixture containing 500 nM RNA probe, 500 nM DNA probe, 250 nM LlCsm effector complex, 1 nM LlCsm6 in a buffer containing 33 mM Tris-acetate pH 7.6 (at 32 °C), 66 mM potassium acetate, 10 mM MnCl_2_, 0–2 mM ATP at 37 °C. The fluorescence was measured on Spectramax ID5 multi-mode microplate reader (Molecular Devices) using dual 480/530 nm and 570/630 nm excitation/emission wavelengths at 1 min intervals. The reactions were performed in triplicates and averaged for the final plots.

### Lysine-specific cross-linking and mass spectrometry analysis

Each of the CTR- and NTR-bound pre-assembled RNP complexes at ~10 nM was separately incubated with 1:1 mixture of deuterated BS3-d4 (bis(sulfosuccinimidyl) 2,2,7,7-suberate-d4) (ThermoFisher Scientific) and non-deuterated BS3-d0 (bis(sulfosuccinimidyl) suberate-d0) (ThermoFisher Scientific) for 60 min. After quenching the cross-linking reactions, the BS3-d0 cross-liked CTR-bound was mixed with the BS3-d4 cross-linked NTR-bound sample and the BS3-d4 cross-liked CTR-bound was mixed with the BS3-d0 cross-linked NTR-bound sample. Both combined samples were resolved on a 10% sodium dodecyl sulfate–polyacrylamide gel electrophoresis gel and the bands corresponding to the cross-linked region were cut, de-stained with 50% aqueous acetonitrile (with 50 mM ammonium bicarbonate), shrunk with acetonitrile, and dried in SpeedVac. Dried gel pieces were then re-hydrated in 10% aqueous acetonitrile with 50 mM ammonium bicarbonate. Trypsin (0.005 μg/μL, ThermoFisher Scientific, catalog #: 90058, Waltham, MA) digestion was performed at 37 °C overnight following 10 min reduction by 0.5 mM DTT (1,4-Dithiothreitol, Sigma-Aldrich, catalog #: 11583786001, St. Louis, MO) at 37 °C. After collecting the supernatant, 0.5% formic acid was added to the residual gel pieces to quench the digestion and further extract tryptic peptides. After collecting the supernatant, the gel pieces were shrunk with acetonitrile and the combined supernatant was dried in SpeedVac.

The tryptic peptides were separated on an Easy-nLC 1200 system (ThermoFisher Scientific, Waltham, MA), ionized by nano-electrospray ionization, and detected by an Orbitrap Exploris 480 mass spectrometer (ThermoFisher Scientific, Waltham, MA). The mobile phases were A (aqueous with 0.1% formic acid) and B (90% acetonitrile aqueous with 0.1% formic acid). The gradient is from 1% to 55% B in 3 hr at a flow rate of 300 nL/min. The eluate was ionized at 2.3 kV and ion transfer tube temperature of 275 °C. The precursor ions were detected with a mass resolution of 120 k, AGC (Automatic Gain Control) of three million tons, and a maximum injection time of 50 ms. The top 15 most abundant precursor ions were subject to tandem MS with a 30% HCD collision Energy, mass resolution of 15k, isolation window of 2 Da, and a maximum injection time of 200 ms. The generated raw data were analyzed with Thermo Proteome Discoverer 2.5 with Crosslink_xlinkx (ThermoFisher Scientific, Waltham, MA)^59^. The software identified cross-linked peptides were then manually checked for assignment.

### In vivo plasmid interference assays

Plasmid interference assays were carried out as previously described^16^. Chemically competent BL21-AI *E. coli* were transformed with pCsm variants and transformants were selected on Miller’s LB broth (Invitrogen) agar supplemented with 34 μg/ml chloramphenicol. Single colonies were cultured in super optimal broth medium (SOB) (BD Difco) and made electrocompetent through successive washes with 10% glycerol. In triplicate, 100 ng of pTrcHis plasmid (with or without transcribed complementary target sequences) were added to 50 μl of competent cells in a 0.2 cm-gap Gene Pulser® electroporation cuvette (BioRad) on ice. Cuvettes were transferred to a Gene Pulser II (BioRad) and pulsed with the following settings: 25 μF capacitance, 2.5 kV, and 200 ohms. Immediately following transformation, 950 μl of super optimal broth with catabolite repression (SOC) (SOB + 20 mM glucose) was used to recover pulsed cells from the cuvette and moved to 1.5 ml microcentrifuge tubes. The tubes were shaken at 200 rpm at 37 °C for 60 min. Serial 10-fold dilutions were made to 10-5 and spot plated onto LB agar containing 100 μg/mL ampicillin and 34 μg/mL chloramphenicol to select for pTrcHis and pCsm, respectively. Plates were imaged after overnight incubation at 37 °C.

### Reporting summary

Further information on research design is available in the Nature Research Reporting Summary linked to this article.

## Supplementary information

Supplementary Information

Reporting Summary

## Data Availability

Cryo-EM maps (coordinates) that support the findings of this study have been deposited to Protein Data Bank with identification codes EMD-22266 (6XN3), EMD-22267 (6XN4), EMD-22269 (6XN7), EMD-22268 (6XN5) for the CTR-43 complex, the CTR-32 complex, the NTR complex, and the apo complex, respectively. Uncropped and unedited gel data that support this are included in Figure S12. A key resources table is available as Table S6.

## Source data

Source Data
