# Supplementary material for: Structural and biochemical characterization of in vivo assembled Lactococcus lactis CRISPR-Csm complex
Source: Commun Biol. 2022 Mar 29;5:279. doi: 10.1038/s42003-022-03187-1 (PMC8964682; doi:10.1038/s42003-022-03187-1)
Supplement: Supplementary file 1 — Supplementary Information [file 42003_2022_3187_MOESM1_ESM.pdf]

SUPPLEMENTARY INFORMATION

Structural and Biochemical Characterization of in vivo Assembled *Lactococcus lactis* CRISPR-Csm Complex

Sagar Sridhara[1], Jay Rai[1], Charlisa Whyms[2], Hemant Goswami[1], Huan He[1], Walter Woodside[3], Michael P Terns[3,4,5] & Hong Li[1,2*]

[1]Institute of Molecular Biophysics, Florida State University, Tallahassee, FL 32306, USA.

[2]Department of Chemistry and Biochemistry, Florida State University, Tallahassee, FL 32306, USA.

[3]Department of Microbiology, University of Georgia, Athens, GA 30602, USA.

[4]Department of Biochemistry and Molecular Biology, University of Georgia, Athens, GA 30602, USA.

[5]Department of Genetics, University of Georgia, Athens, GA 30602, USA.

[*]Corresponding author: hong.li@fsu.edu

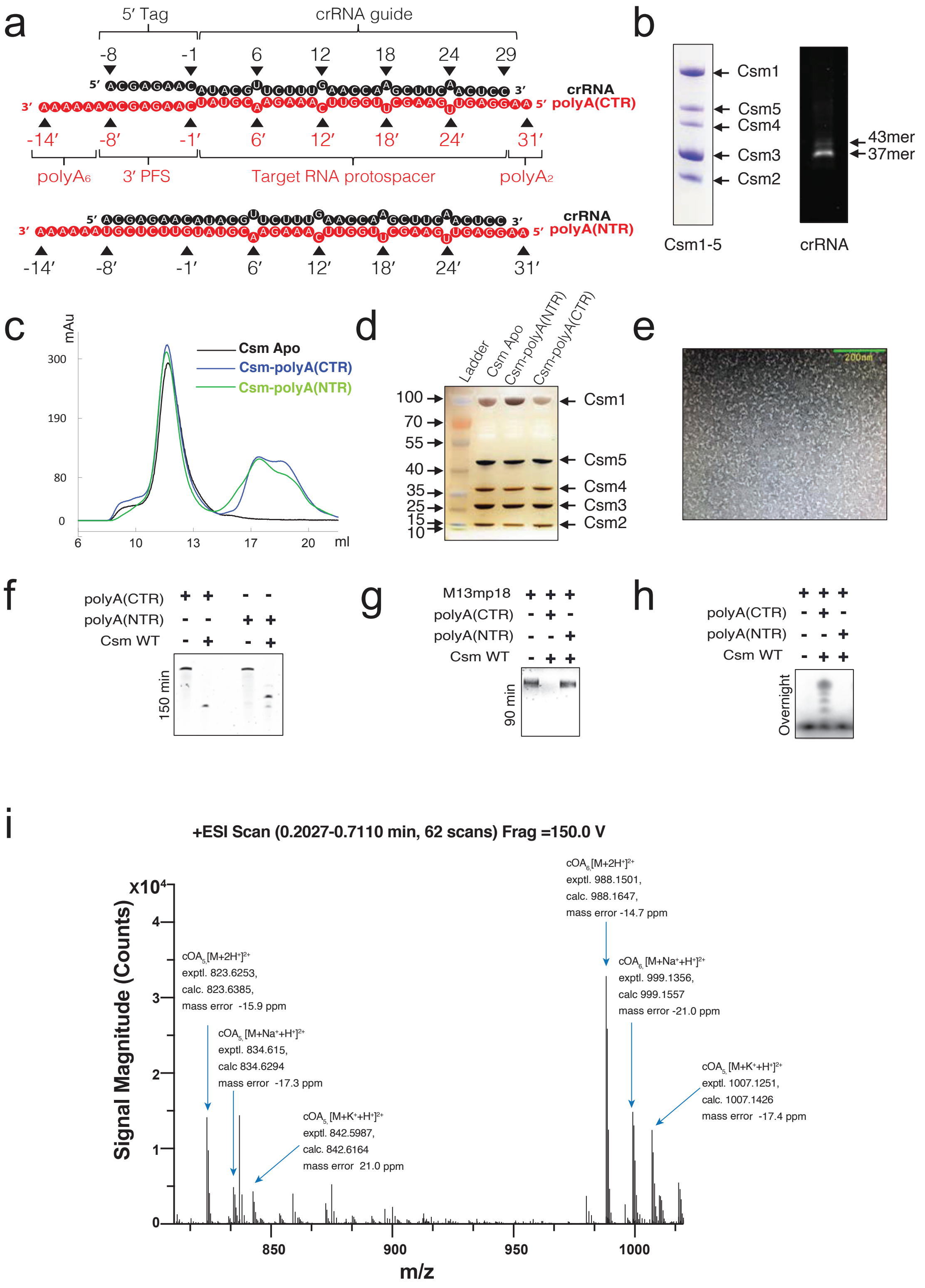

a
5′ Tag
crRNA guide
-8
-1
6
12
18
24
29
crRNA
polyA(CTR)
-14′
-8′
-1′
6′
12′
18′
24′
31′
polyA6
3′ PFS
Target RNA protospacer
polyA2
crRNA
polyA(NTR)
b
Csm1
Csm5
Csm4
Csm3
Csm2
Csm1-5
43mer
37mer
crRNA
c
mAu
Csm Apo
Csm-polyA(CTR)
Csm-polyA(NTR)
ml
d
Ladder
Csm Apo
Csm-polyA(NTR)
Csm-polyA(CTR)
Csm1
Csm5
Csm4
Csm3
Csm2
e
200nm
f
polyA(CTR)
polyA(NTR)
Csm WT
150 min
g
M13mp18
polyA(CTR)
polyA(NTR)
Csm WT
90 min
h
polyA(CTR)
polyA(NTR)
Csm WT
Overnight
i
+ESI Scan (0.2027-0.7110 min, 62 scans) Frag =150.0 V
Signal Magnitude (Counts)
x10^4
m/z
cOA5 [M+2H+]2+ exptl. 823.6253, calc. 823.6385, mass error -15.9 ppm
cOA5 [M+Na++H+]2+ exptl. 834.615, calc 834.6294 mass error -17.3 ppm
cOA5 [M+K++H+]2+ exptl. 842.5987, calc. 842.6164 mass error 21.0 ppm
cOA6 [M+2H+]2+ exptl. 988.1501, calc. 988.1647, mass error -14.7 ppm
cOA6 [M+Na++H+]2+ exptl. 999.1356, calc 999.1557 mass error -21.0 ppm
cOA5 [M+K++H+]2+ exptl. 1007.1251, calc. 1007.1426 mass error -17.4 ppm

Figure S1. Cryo-EM sample preparation and activity tests on target RNA. Related to Figures 1, 2, 3 and 4. (a) Schematic representation and nomenclature of crRNA-polyA(CTR) and crRNA-polyA(NTR) duplexes. The target RNAs used in the cryo-EM samples contained 6-nt and 2-nt polyA extensions at 3'- and 5'-ends respectively. (b) Sodium dodecyl sulfate - polyacrylamide gel electrophoresis (SDS-PAGE) and denaturing Urea PAGE gel images showing the quality of *L. lactis* ribonucleoprotein (RNP) used to make target-free and target-bound complexes for structural studies. The purified RNP contained all five proteins forming the effector complex (Csm1-5) and two species of crRNA (major 37mer species and minor 43mer species). (c) Overlay of size-exclusion chromatograms of no-target (apo), polyA(CTR) and polyA(NTR)-bound Csm complexes in black, blue and green colors respectively. (d) Silver stain profiles of no-target (apo), polyA(CTR) and polyA(NTR)-bound Csm complexes showing the presence of all 5 Csm subunits in all sample preparations. The loaded amounts are too low for crRNA to be visible. (e) Negative stain showing homogenous distribution of LlCsm particles during sample screening. The image was collected using FEI CM120 Biotwin transmission electron microscope. (f) *In vitro* RNase cleavage assay showing that polyA(CTR) and polyA(NTR) used for structure determination is similar to RNase activity by Csm WT on CTR and NTR respectively. (g) *In vitro* DNase cleavage assay showing that polyA(CTR) and polyA(NTR) used for structure determination is similar to DNase activity by Csm WT on CTR and NTR respectively. (h) *In vitro* cOA synthesis assay with polyA(CTR) and polyA(NTR) as the stimulators that were used for structure studies. (i) Mass spectra of cOA synthesis products. Identified cOA species are indicated arrows with expected and calculated masses, charge states, and mass errors indicated.

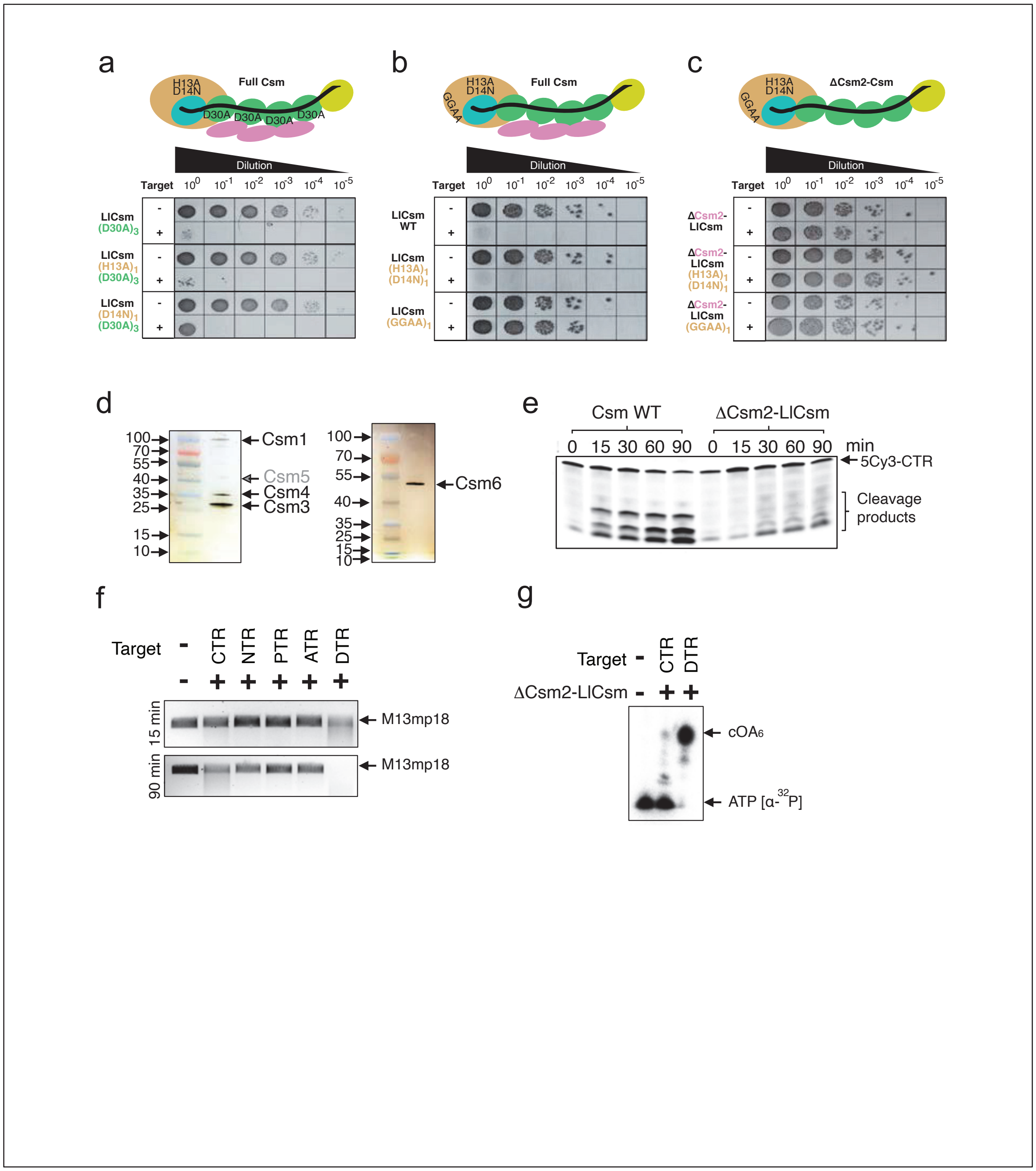

Figure S2. Assessment of functionality of the wild-type and ΔCsm2-LlCsm complexes using cell plasmid interference and biochemical assays. Related to Figures 2, 3 and 4, Table S1. (a) Plasmid interference activities of full LlCsm complex harboring Csm1 and/or Csm3 mutations. (b) Plasmid interference activities of full LlCsm harboring Csm1 HD

mutation or Csm1 palm2 mutation. (c) Plasmid interference activities of ΔCsm2-LlCsm harboring Csm1 HD mutation or Csm1 palm2 mutation. (d) Silver stain profiles of the co-purified ΔCsm2-LlCsm complex (left) and His-tagged LlCsm6 protein (right). The ΔCsm2-LlCsm complex comprises of intact Csm1, Csm4 and Csm3. Csm5 that copurified with His-tagged Csm3 is low but present. (e) *In vitro* RNA cleavage assay by the WT and ΔCsm2-LlCsm complex. The RNase reaction products of 5'-Cy3-CTR were analyzed at time points: 0 min, 15 min, 30 min, 60 min and 90 min. (f) *In vitro* DNA cleavage assay by the ΔCsm2-LlCsm complex. M13mp18 was used as substrate and the reaction products were analyzed at time points 15 min and 90 min. (g) *In vitro* cOA synthesis assay by the ΔCsm2-LlCsm complex. Radiolabeled α-^{32}P-ATP was used as a substrate and the reactions were incubated overnight.

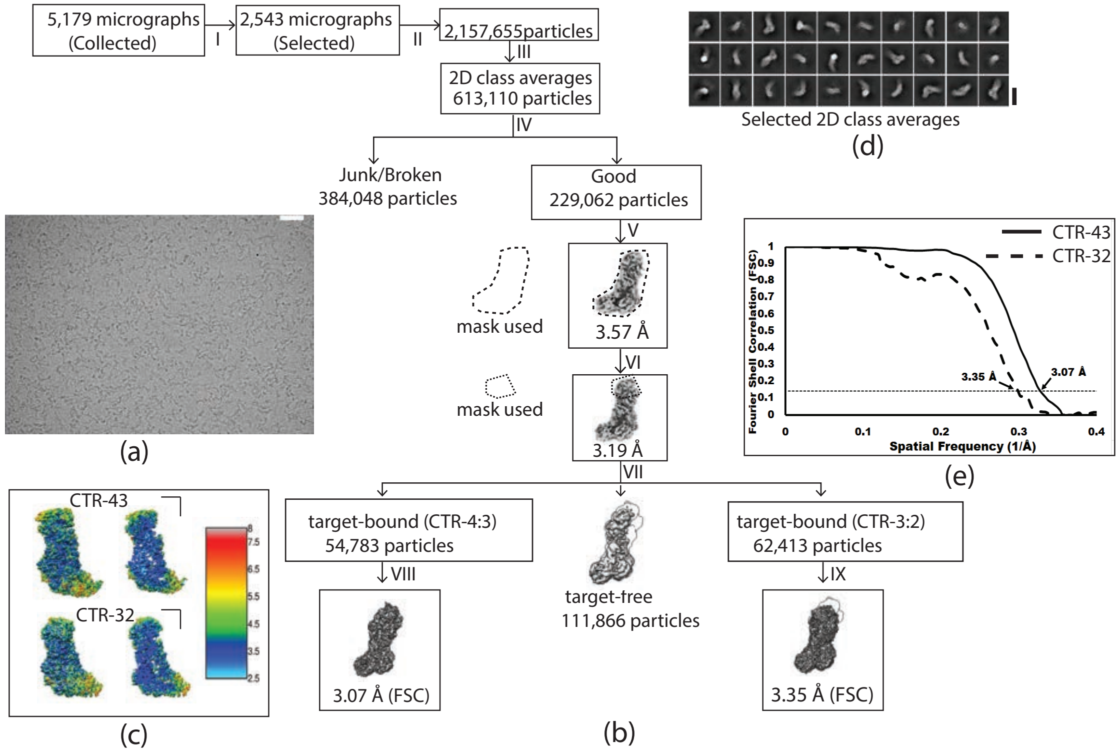

Figure S3. Cryo-EM data processing and refinement flow chart of LlCsm-CTR-43 and LlCsm-32 complex. Related to Figure 2. (a) Raw micrograph (scale bar 20 nm) (b) Image processing flowchart: In total 5,179 micrographs were collected. 2,543 were chosen for the frame alignment. 2,15,655 particles were extracted using Relion 3.0 (1) and imported into cryoSPARC software (2) to perform multiple rounds of 2D analysis. After 2D analysis, 613,110 particles were selected and imported to Relion 3.0 to perform 3D classification that resulted in 229,062 particles which further refine to 3.57 Å resolution and further per particles CTF refine followed by autorefine improved the resolution to 3.19 Å. 3D classification was performed using mask on the top part (Csm5), which sorted out the particles into three classes. First class contain four copies of Csm3 densities and three copies of Csm2 densities (CTR-43). Second class lack Csm2 densities and target RNA densities. Third class contain the three copies of Csm3 densities and two copies of Csm2 densities (CTR-32). 54,783 particles from first class

was imported into CisTEM software (3) and further refined to obtain 3.07 Å resolution. 62,413 particles from third class were further refined using per particle CTF refine and autorefine that resulted in 3.5 Å. (c) Local resolution estimated by Resmap. (d) Selected 2D class averages (scale bar 15 nm). (e) FSC curves.

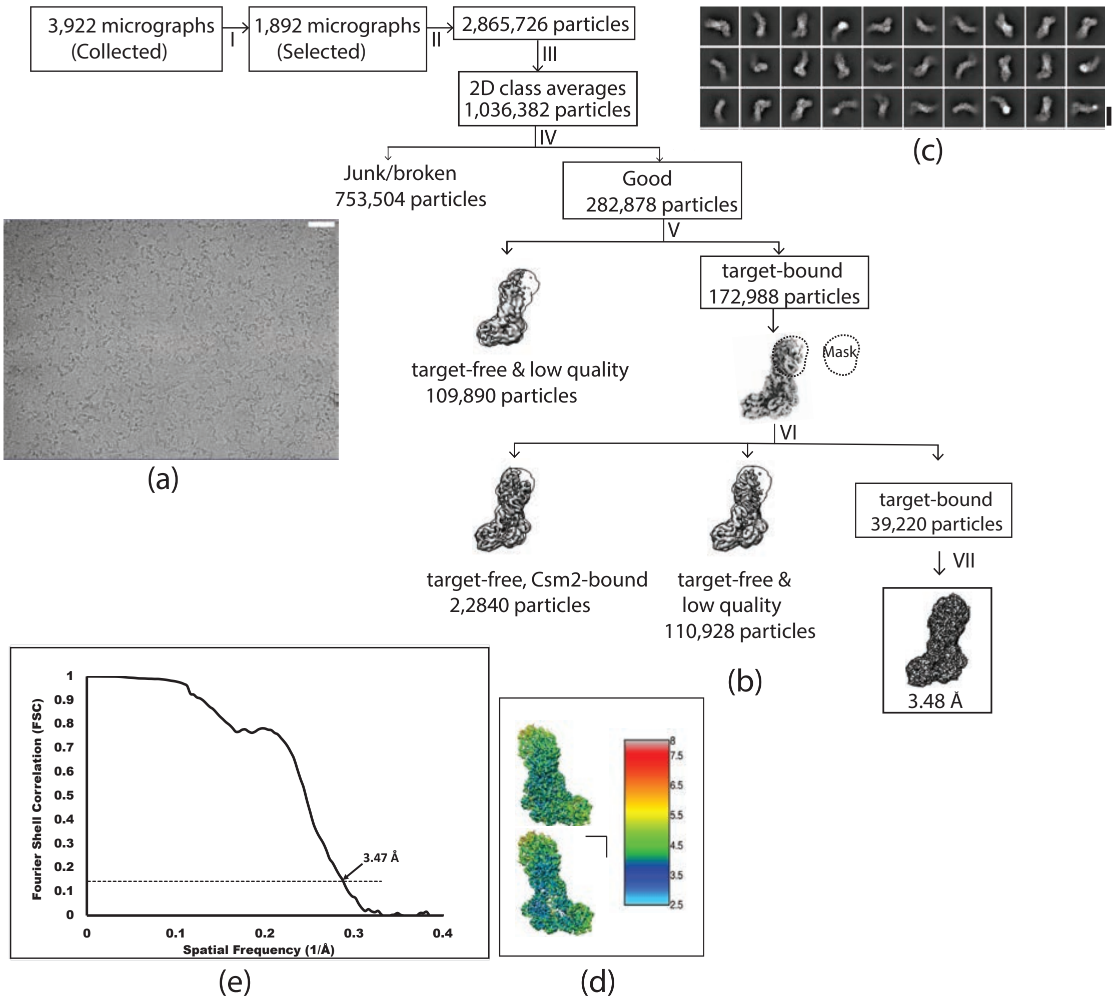

Figure S4. Cryo-EM data processing and refinement flow chart of LlCsm-NTR complex. Related to Figure 3. (a) Raw micrograph (Scale bar 50 nm) (b) Image processing flow chart: In total 3,922 micrographs were collected. 1,892 were chosen for the frame alignment. 2,86,726 particles were extracted using RELION 3.0 (4) and imported to cryoSPARC software (2) to perform multiple rounds of 2D analysis. After 2D analysis, 1,036,382 particles were selected to perform 3D classification that resulted in 282,878 particles followed by another round of 3D classifications. The particles with target RNA were pooled together and imported into RELION for classification based on Csm5 using mask on the top part (Csm5) resulting in three classes.

The two classes showed no density of target RNA and were discarded. The third class with target density was further refined with ctf refine that resulted in 3.48 Å resolution. (c) Local resolution estimated by Resmap. (d) Selected 2D class averages (scale bar 15 nm). (e) FSC curve.

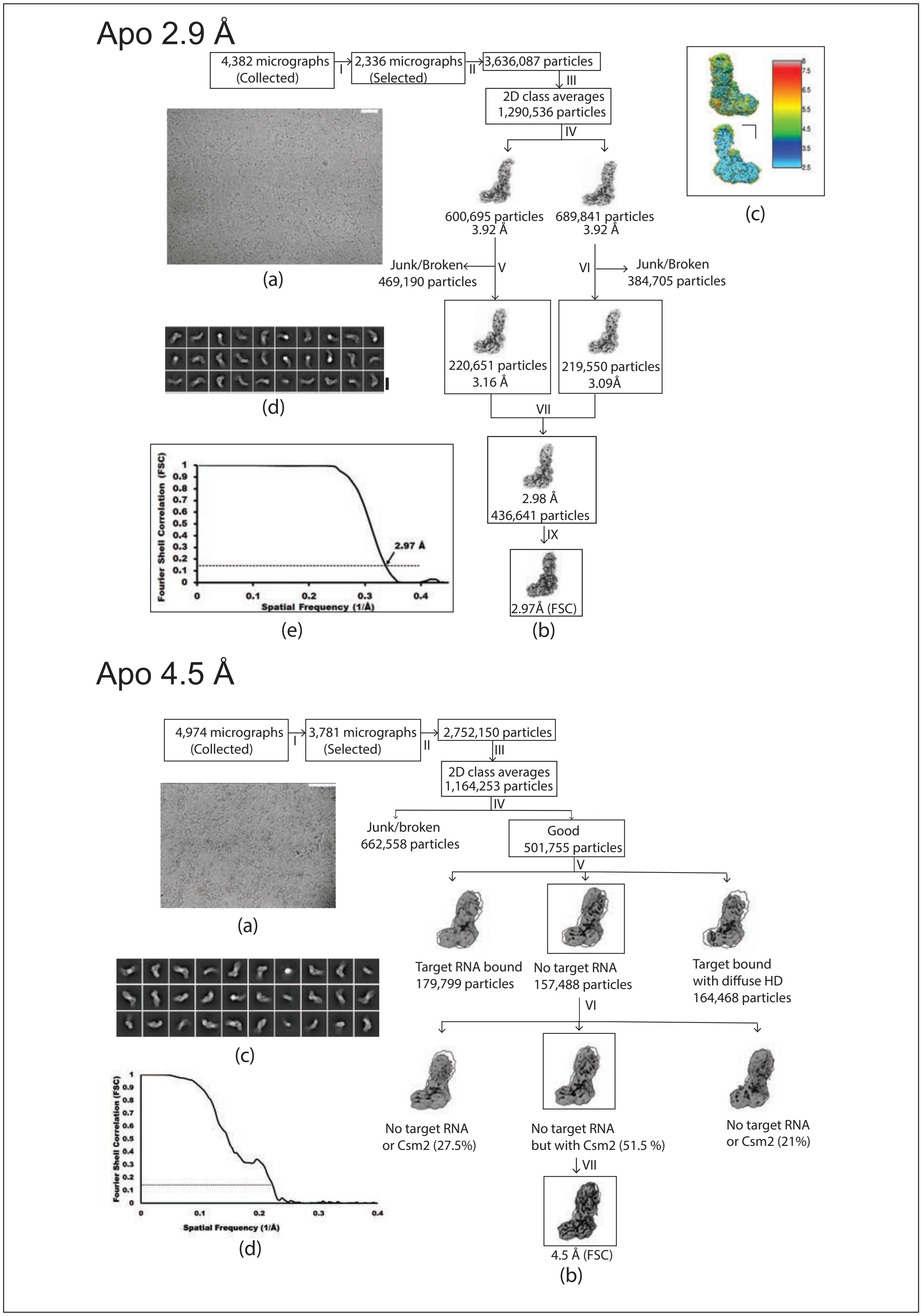
Apo 2.9 Å
4,382 micrographs (Collected)
I
2,336 micrographs (Selected)
II
3,636,087 particles
III
2D class averages 1,290,536 particles
IV
600,695 particles 3.92 Å
689,841 particles 3.92 Å
Junk/Broken 469,190 particles
V
VI
Junk/Broken 384,705 particles
220,651 particles 3.16 Å
219,550 particles 3.09Å
VII
2.98 Å 436,641 particles
IX
2.97Å (FSC)
Fourier Shell Correlation (FSC)
Spatial Frequency (1/Å)
2.97 Å
(a)
(b)
(c)
(d)
(e)
Apo 4.5 Å
4,974 micrographs (Collected)
I
3,781 micrographs (Selected)
II
2,752,150 particles
III
2D class averages 1,164,253 particles
IV
Junk/broken 662,558 particles
Good 501,755 particles
V
Target RNA bound 179,799 particles
No target RNA 157,488 particles
Target bound with diffuse HD 164,468 particles
VI
No target RNA or Csm2 (27.5%)
No target RNA but with Csm2 (51.5 %)
No target RNA or Csm2 (21%)
VII
4.5 Å (FSC)
Fourier Shell Correlation (FSC)
Spatial Frequency (1/Å)
(a)
(b)
(c)
(d)

Figure S5. Cryo-EM data processing and refinement flow chart of LlCsm-apo complexes. Related to Figure 4.

Top, Apo 2.9 Å complex. (a) Raw micrograph (Scale bar 50 nm). (b) Local resolution estimated by Resmap. (c) Image processing flow chart: In total 4,382 micrographs were collected. 2,336 were chosen for the frame alignment. 3,636,687 particles were extracted using RELION 3.0 (4) and imported to cryoSPARC software (2) to perform multiple rounds of 2D analysis. After 2D analysis, 1,290,536 particles were selected and imported to RELION 3 (1) to perform 3D classification that resulted in 436,641 good particles which were used for per particle CTF refine followed by autorefine yielding the resolution of 2.98 Å. Furthermore, the particles were imported in cisTEM which resulted in the resolution of 2.97 Å. (d) Selected 2D class averages (scale bar 15 nm). (e) FSC curve.

Bottom, Apo 4.5 Å complex with analogous analysis as top. (a) Raw micrograph (Scale bar 50 nm). (b) Image processing flow chart; (c) Selected 2D class averages (scale bar 15 nm). (d) FSC curve.

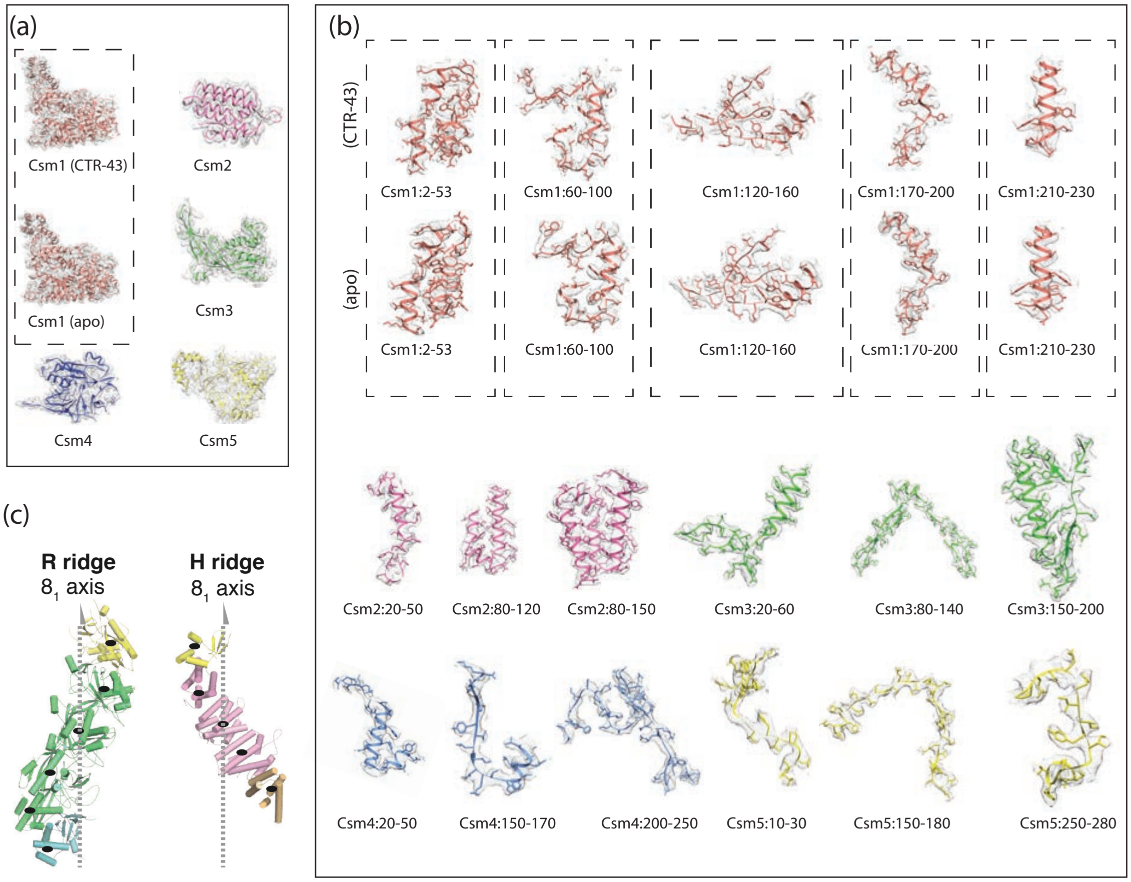

Figure S6. Density maps superimposed with finally refined models. Related to Figures 2, 3 and 4. (a) The density map of each subunit is from the CTR-43 structure while that of Csm1 from the apo structure is also shown for comparison. (b) Focused views of the density for selected regions are shown. The densities of Csm1 are shown for both CTR-43 and apo structures for comparison. (c) Isolated R and H ridges with the 8-fold screw axis indicated.

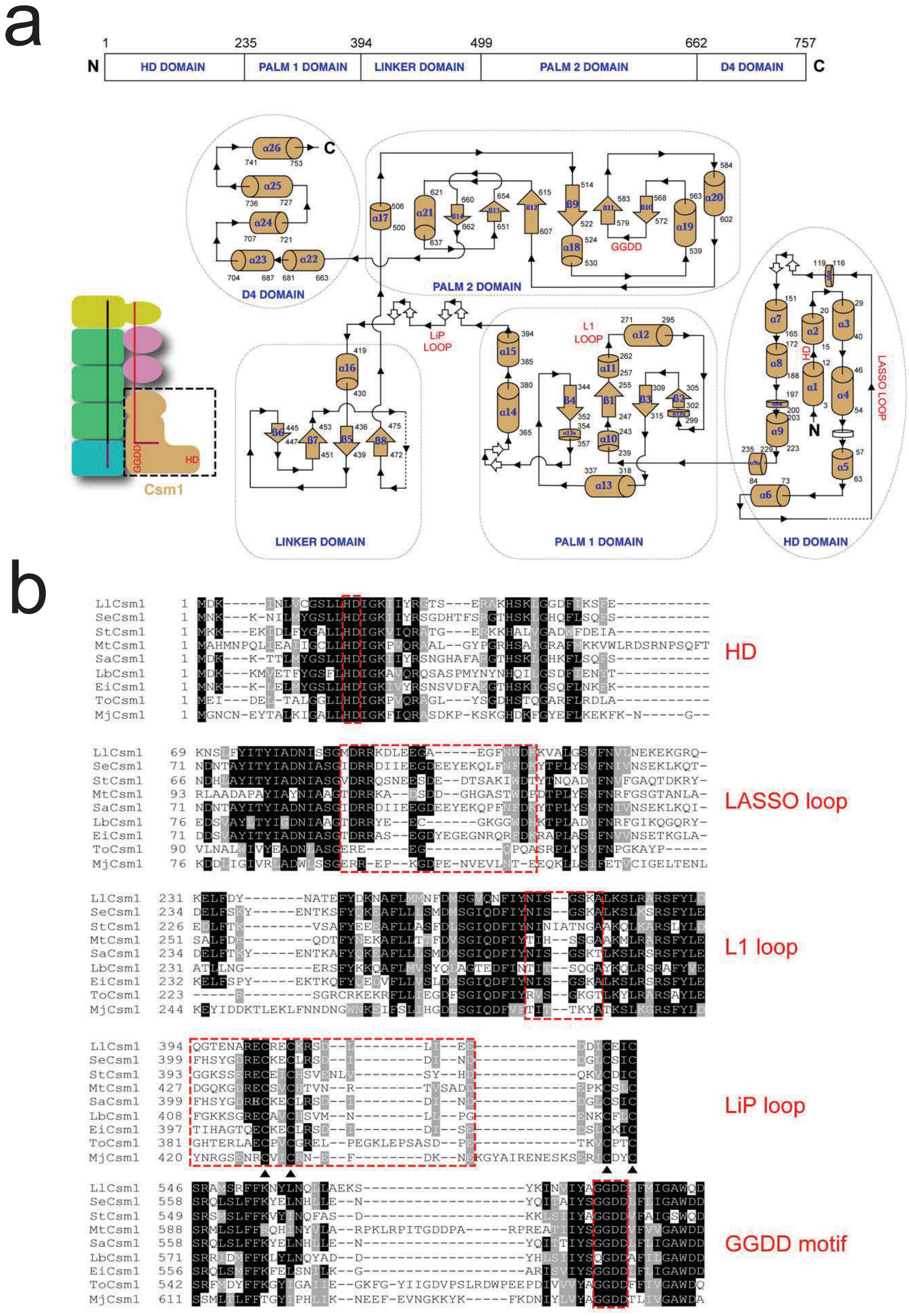
a
N
HD DOMAIN
PALM 1 DOMAIN
LINKER DOMAIN
PALM 2 DOMAIN
D4 DOMAIN
C
1
235
394
499
662
757
Csm1
GGDD
HD
D4 DOMAIN
PALM 2 DOMAIN
LINKER DOMAIN
PALM 1 DOMAIN
HD DOMAIN
LiP LOOP
L1 LOOP
LASSO LOOP
b
LlCsm1
SeCsm1
StCsm1
MtCsm1
SaCsm1
LbCsm1
EiCsm1
ToCsm1
MjCsm1
HD
LASSO loop
L1 loop
LiP loop
GGDD motif

Figure S7. Domain organization, topology and multiple sequence alignment of LlCsm1. Related to Figures 2, 3 and 4. (a) Topology map of LlCsm1 composed of N-terminal HD domain, palm1 domain, linker domain, palm2 domain and C-terminal D4 domain. The DNase HD catalytic site, the GGDD motif constituting the cOA synthesis site and the loops important in dynamic functioning: Lasso loop, L1 loop and LiP loop are highlighted. (b) Multiple sequence alignment of *Lactococcus lactis* Csm1 (LlCsm1) with bacterial Csm1 orthologs from *Staphylococcus epidermidis* (SeCsm1), *Streptococcus thermophilus* (StCsm1), *Mycobacterium tuberculosis* (MtCsm1), *Staphylococcus aureus* (SaCsm1), *Lactobacillus delbrueckii* subsp. *bulgaricus* (LbCsm1), *Enterococcus italicus* (EiCsm1) and archaeal orthologs from *Thermococcus onnurineus* (ToCsm1), *Methanocaldococcus jannaschii* (MjCsm1). The red dashed boxes highlight highly conserved HD residues, sequence-variable lasso loop, L1 loop, LiP loop and conserved GGDD motifs. The black triangles indicate four strongly conserved cysteine residues forming the Zinc-finger motif in Csm1 orthologs.

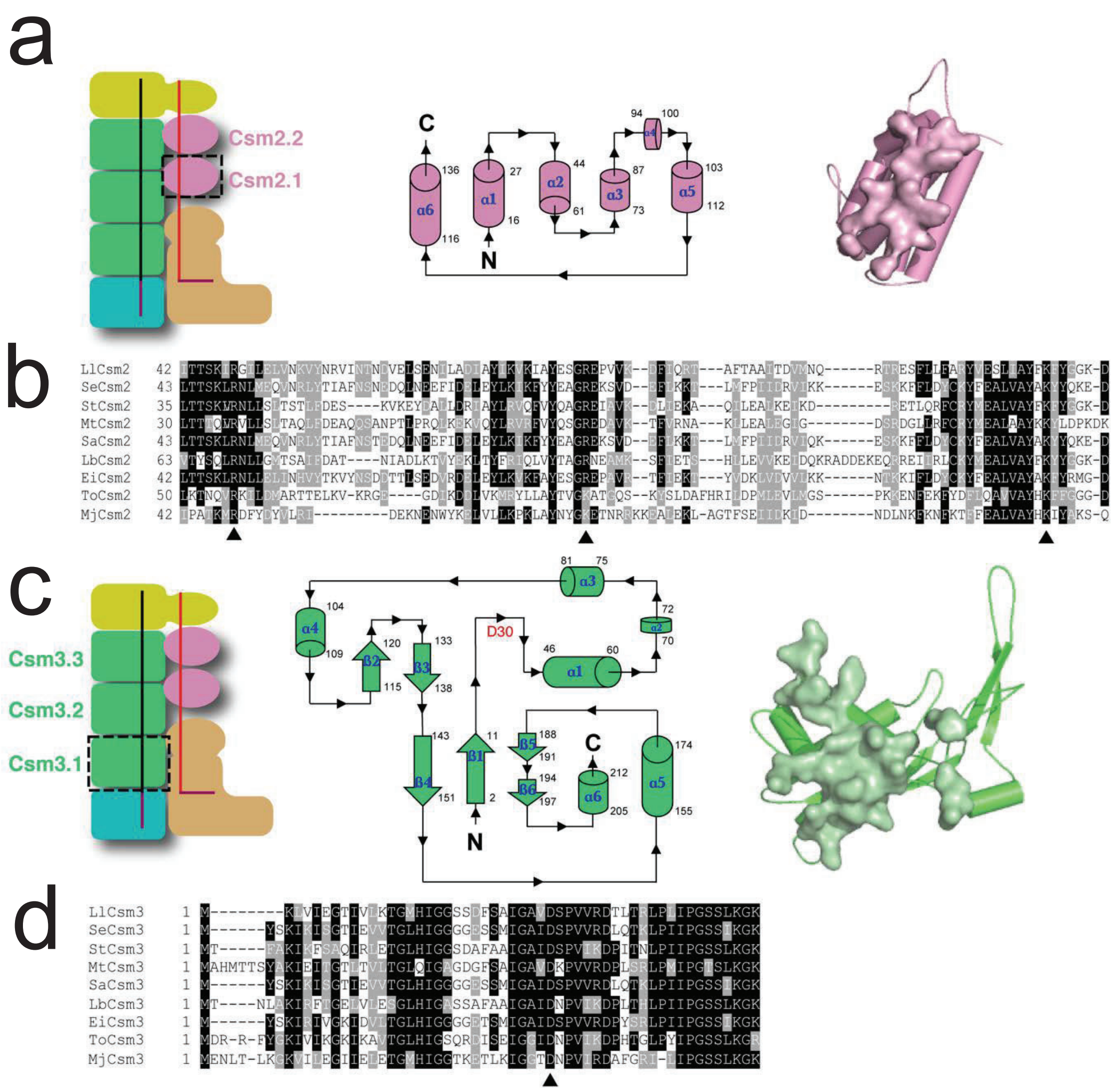

Figure S8. Topology, multiple sequence alignment and subunit interfaces of LlCsm2 and LlCsm3. Related to Figure 5. (a) Topology map of helical LlCsm2. Interface residues of Csm2 are identified to be 4 Å from its neighboring Csm2 subunit and are shown in surface representation. (b) Multiple sequence alignment of *Lactococcus lactis* Csm2 (LlCsm2) with bacterial Csm2 orthologs from *Staphylococcus epidermidis* (SeCsm2), *Streptococcus thermophilus* (StCsm2), *Mycobacterium tuberculosis* (MtCsm2), *Staphylococcus aureus* (SaCsm2), *Lactobacillus delbrueckii* subsp. *bulgaricus* (LbCsm2), *Enterococcus italicus*

(EiCsm2) and archaeal orthologs from *Thermococcus onnurineus* (ToCsm2), *Methanocaldococcus jannaschii* (MjCsm2). Important residues are highlighted by black triangles. (c) Topology map of LlCsm3. The location of RNase catalytic site D30 is highlighted in red. Interface residues of Csm3 are identified to be 4 Å from its neighboring Csm3 subunit and are shown in surface representation. (d) Multiple sequence alignment of *Lactococcus lactis* Csm3 (LlCsm3) with bacterial Csm3 orthologs from *Staphylococcus epidermidis* (SeCsm3), *Streptococcus thermophilus* (StCsm3), *Mycobacterium tuberculosis* (MtCsm3), *Staphylococcus aureus* (SaCsm3), *Lactobacillus delbrueckii* subsp. *bulgaricus* (LbCsm3), *Enterococcus italicus* (EiCsm3) and archaeal orthologs from *Thermococcus onnurineus* (ToCsm3), *Methanocaldococcus jannaschii* (MjCsm3). The strongly conserved Aspartate residue important for target RNA cleavage is highlighted in black triangle.

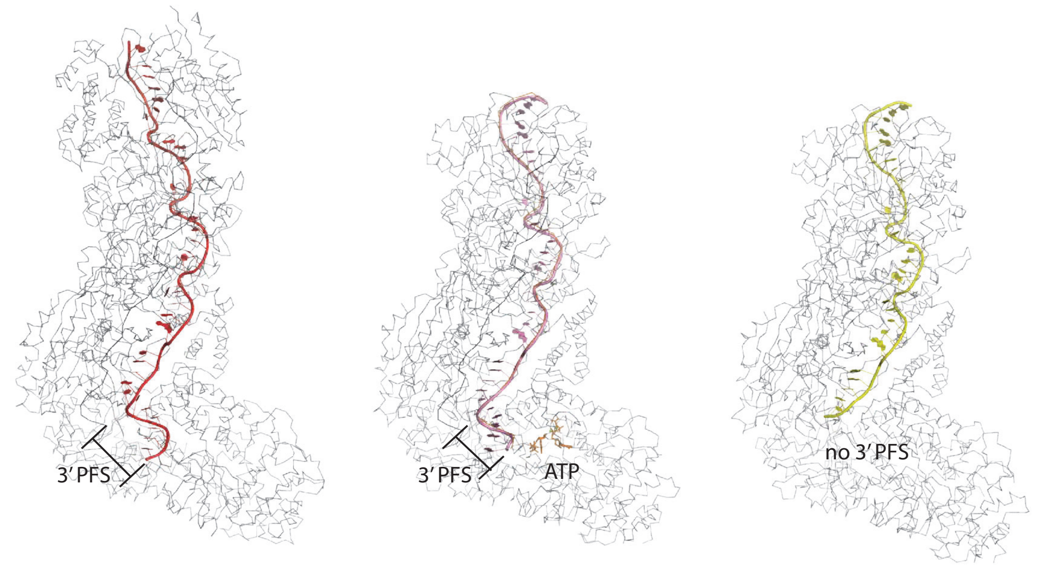

Figure S9. Comparison of 3' PFS structures between LlCsm and StCsm. Related to Figures, 2, 3, & 4. The target RNA in each of the three complexes is colored red (LlCsm), pink (StCsm, 6IG0), or yellow (StCsm, 6IFY), respectively. No 3' PFS was observed in StCsm complex in absence of ATP or its non-bydrolysable analogs.

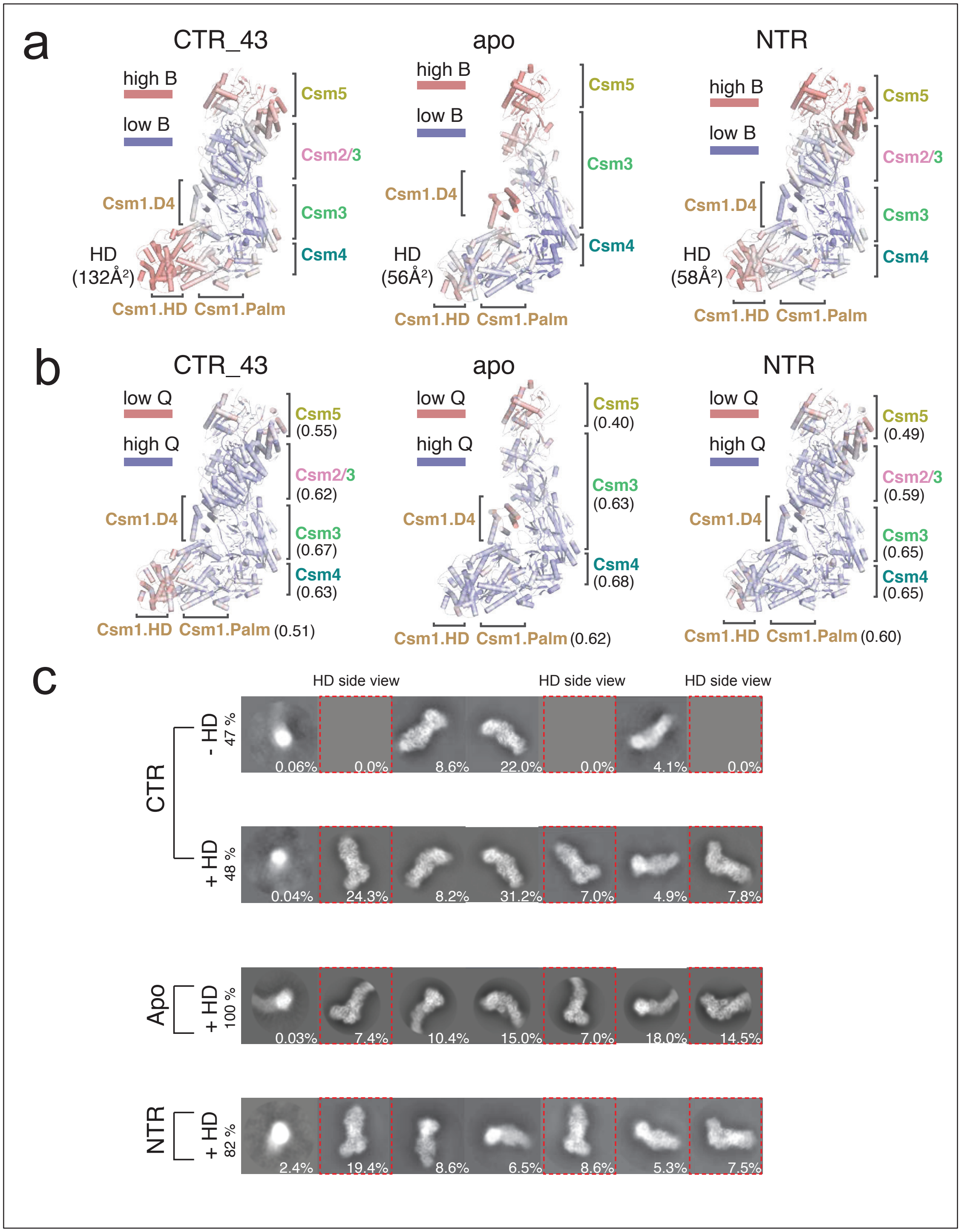

Figure S10. Flexibility of the three Llcsm complexes indicated by model B factors and 2D class average analyses. Related to Figure 6. (a) B factors of each complex are plotted on the refined models in a color gradient with blue being low and red being high values. Location of the subunits or domains of Csm1 are labeled and the average B factor of the HD domain is included in the parenthesis for each complex.

(b) Q scores of each complex are plotted on the refined models in a color gradient with blue being high and red being low values. Location of the subunits or domains of Csm1 are labeled. Average Q-scores for all subunits are included in the paranthesis. (c) 2D class averages of particles used for reconstruction of the CTR-43, Apo, and NTR complexes. For each complex, particles were 3D sorted using a mask around the HD domain followed by 2D class averaging. CTR-43 particles showed two major classes, one with the HD domain and one lacking the HD domain, while NTR and the Apo particles had primarily the class with HD domain. 2D averages were obtained for the CTR-43 particles with HD domain (+HD) and those without HD domain (-HD) and for Apo and NTR particles. The percent of each 2D class with respect to total is indicated. A large number of CTR-43 particles lack the sideviews of the HD domain.

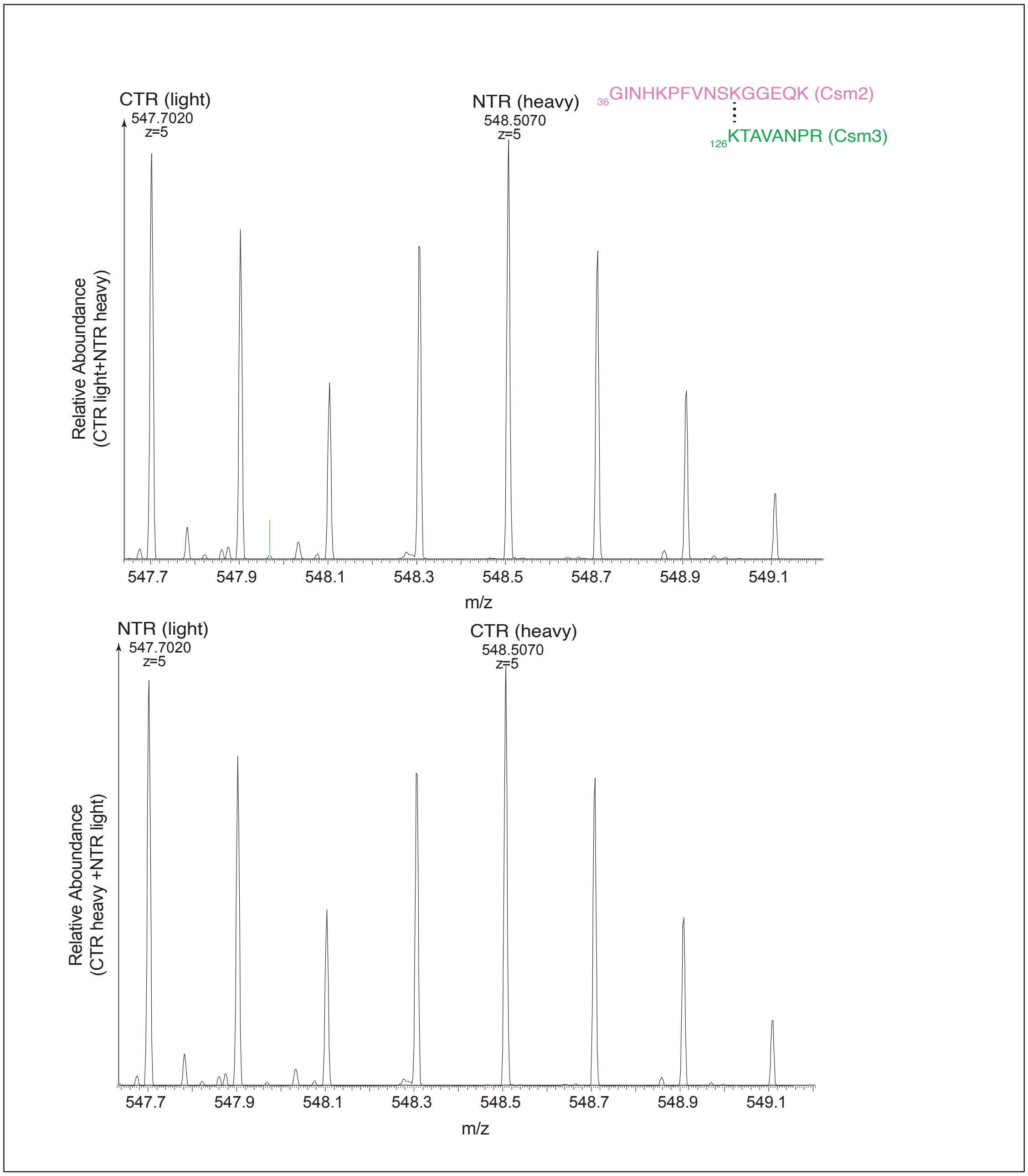

Figure S11. Example spectra of lysine-specific crosslinks indicating similar abundance between the CTR- and the NTR-bound LlCsm complexes. Related Figure 6. The deuterated BS3 (bis(sulfosuccinimidyl) 2,2,7,7-suberate)-d4 is designated as heavy and the non-deuterated BS3-d0 is designated as light, respectively. The peptide sequences for Csm3 and Csm2

matching the identified crosslinks are indicated in the same color scheme used for structural models. The mass and the ion state are indicated to the known accuracy of the instrument.

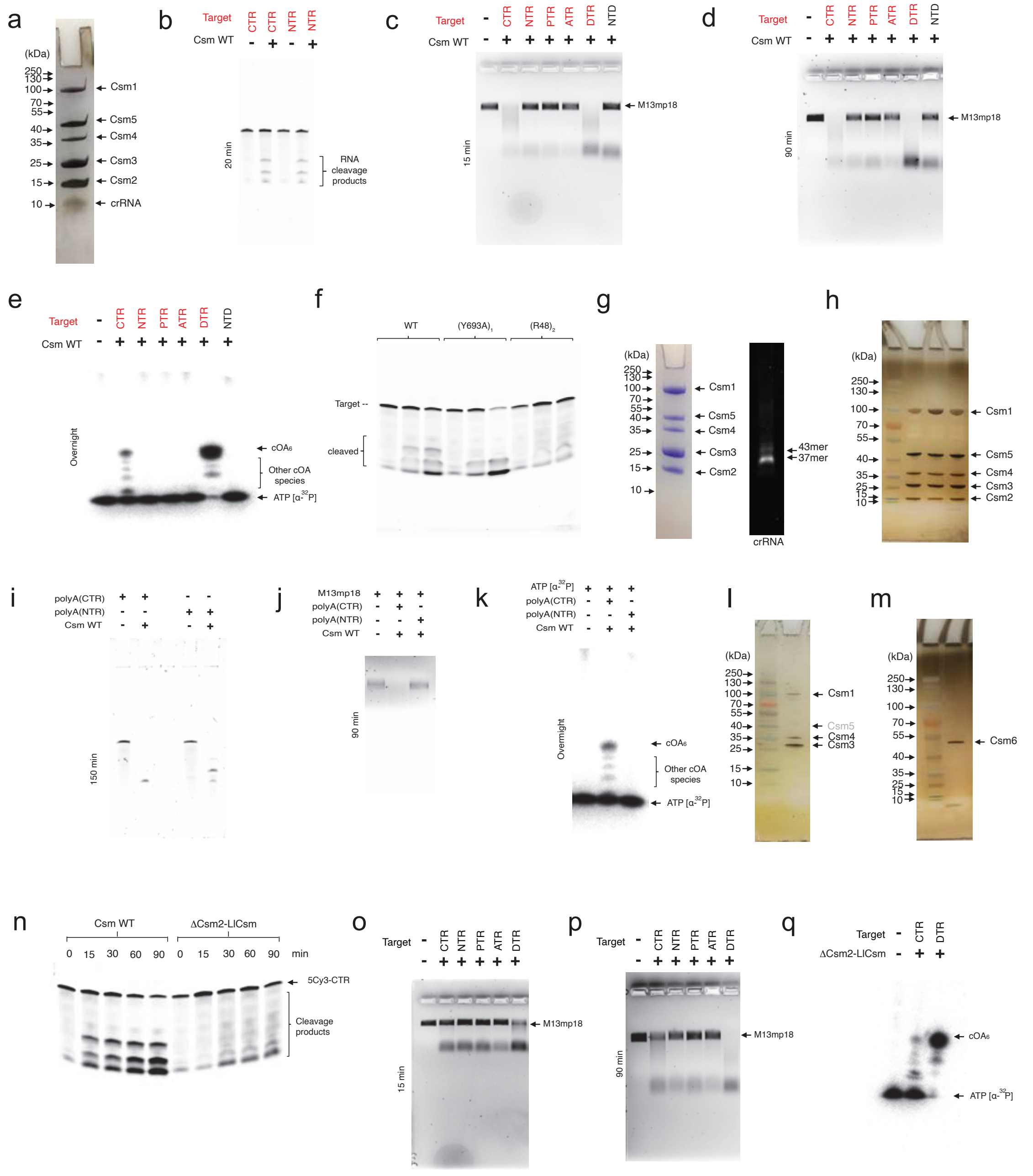

Figure S12. Uncropped Images. Related to Figures 1, 5, S1 and S2. (a) Silver stain profile shown in Figure 1D (b) *In vitro* RNA cleavage assay shown in Figure 1E (c) *In vitro* DNA cleavage assay shown in Figure 1F (top) (d) *In vitro* DNA cleavage assay shown in Figure 1F (bottom) (e) Thin layer chromatography (TLC) analysis of cOA synthesis shown in Figure 1G

(f) *In vitro* RNA cleavage assay shown in Figure 5C (g) SDS-PAGE and denaturing Urea-PAGE gel images shown in Figure S1B (h) Silver stain profile shown in Figure S1D (i) *In vitro* RNA cleavage assay shown in Figure S1F (j) *In vitro* DNA cleavage assay shown in Figure S1G (k) *In vitro* cOA synthesis assay shown in Figure S1H (l & m) Silver stain profiles shown in Figure S2D (n) *In vitro* RNA cleavage assay shown in Figure S2E (o) *In vitro* DNA cleavage assay shown in S2F (top) (p) *In vitro* DNA cleavage assay shown in S2F (bottom) (q) *In vitro* cOA synthesis assay shown in S2G.

Table S1: Compilation of observations of plasmid interference assay and assessment of functionality of variants of LlCsm complex. Related to Figures 1 and S2. Wild type LlCsm harboring non-cognate crRNA (WT/NCR), wild type LlCsm harboring cognate crRNA (WT/CCR), LlCsm Csm3 Asp30Ala mutant (LlCsm $(D30A)_3$), LlCsm Csm1 His13Ala Csm3 Asp30Ala double mutant (LlCsm $(H13A)_1(D30A)_3$), LlCsm Csm1 Asp14Asn Csm3 Asp30Ala double mutant (LlCsm $(D14N)_1(D30A)_3$), LlCsm Csm1 His13Ala Asp14Asn double mutant (LlCsm $(H13A)_1(D14N)_1$), LlCsm Csm1 $(GGDD)_{574\text{-}577}$ to $(GGAA)_{574\text{-}577}$ mutant (LlCsm $(GGAA)_1$), Csm2-deleted LlCsm complex (ΔCsm2-LlCsm), Csm2-deleted LlCsm complex harboring Csm1 His13Ala Asp14Asn mutations (ΔCsm2-LlCsm $(H13A)_1(D14N)_1$) and Csm2-deleted LlCsm complex harboring Csm1 $(GGDD)_{574\text{-}577}$ to $(GGAA)_{574\text{-}577}$ mutation (ΔCsm2-LlCsm $(GGAA)_1$) were tested.

**LlCsm variant**	**Plasmid Interference**
WT/NCR	NO
WT/CCR	YES
LlCsm $(D30A)_3$	YES
LlCsm $(H13A)_1(D30A)_3$	YES
LlCsm $(D14N)_1(D30A)_3$	YES
LlCsm $(H13A)_1(D14N)_1$	YES
LlCsm $(GGAA)_1$	NO
ΔCsm2-LlCsm	NO
ΔCsm2-LlCsm $(H13A)_1(D14N)_1$	NO
ΔCsm2-LlCsm $(GGAA)_1$	NO

Table S2: Statistics of cryo-EM data processing and model refinement. Related to Figures 2, 4 and 5, Table S5.

**Data acquisition and processing parameters**	**CTR_4:3 complex**	**CTR_3:2 complex**	**Apo complex**	**NTR complex**
Microscope	Titan Krios	Titan Krios	Titan Krios	Titan Krios
Detector	Gatan K3	Gatan K3	Gatan K3	Gatan K3
Voltage	300 kV	300 kV	300 kV	300 kV
Electron Source	Field Emission Gun	Field Emission Gun	Field Emission Gun	Field Emission Gun
Collecting Mode	Counting	Counting	Counting	Counting
Dose Rate ($e^-/Å^2$)	60.07	60.07	61.51	60.14
Defocus range (μm)	-1.3 to -2.8	-1.3 to -2.8	-1.3 to -2.8	-1.4 to 3
Nominal Magnification	81000X	81000X	81000X	81000X
Frames collected per exposure	75	75	74	70
Framealingment Software	MotionCor2	MotionCor2	MotionCor2	MotionCor2
CTF parameter estimation Software	Gctf	Gctf	Gctf	Gctf
Total number of raw images collected	5,179	5,179	4,382	3,922
Number of images used for particle picking	2,543	2,543	2,336	1,892
Initial particles picked	2,157,655	2,157,655	3,636,087	2,865,725
2D classification software	Cryosparc (2)	Cryosparc (2)	Cryosparc (2)	Cryosparc (2)
Final reconstruction software	Cistem (3)	Relion 3.1 (4)	Cistem (3)	Relion 3.1 (4)
Applied symmetry	C1	C1	C1	C1
Number of particles contributed for the final reconstruction	54,783	62,413	436,641	39,220
Resolution method	FSC 0.143 cut-off	FSC 0.143 cut-off	FSC 0.143 cut-off	FSC 0.143 cut-off
Map resolution (Å)	3.07	3.35	2.97	3.47
Local resolution determining Software	Resmap	Resmap	Resmap	Resmap
Map Visualization software	Pymol/Chimera/ Chimera X/ Coot (5-7)	Pymol/Chimera/ Chimera X/ Coot (5-7)	Pymol/Chimera/ Chimera X/ Coot (5-7)	Pymol/Chimera/ Chimera X/ Coot (5-7)
Deposit EMDB code	22266	22267	22268	22269
**Refinement parameters**				
CC (map_model) (mask)	0.80	0.78	0.80	0.80
RMSD (Bond lengths/Bond angles)	0.007/1.142	0.009/1.235	0.007/1.115	0.007/1.097
Ramachandran plot (%) (Outlier/Allowed/Favored)	0.00/7.35/92.65	0.14/10.04/89.83	0.00/7.41/92.59	0.04/7.09/92.87
Cβ Outliers (%)	0.00	0.00	0.00	0.00
MolProbity Score	1.83	2.09	1.78	1.76
Clash score	6.59	10.35	5.72	5.58
Rotamer outliers (%)	0.04	0.00	0.06	0.09
ADP (B-fator)<br>Protein (min/mask/mean)<br>Nucleotide (min/mask/mean)	22049<br>23.14/148.15/68.34<br>36.29/133.21/55.59	19273<br>87/812.35/214.36<br>106.85/205.33/152.09	17400<br>21.66/128.16/58.25<br>25.43/99.71/50.11	22209<br>14.13/102.20/41.28<br>14.85/132.12/38.85
dFSC model (0/0.143/0.5)	3.0/3.1/3.5	2.9/3.1/3.7	3.0/3.0/3.3	2.9/3.0/3.5
Deposit PDB code	6XN3	6XN4	6XN5	6XN7

Table S3: Nucleic acid sequences and oligonucleotides used in this study. Related to Figures 1, 4, 5 S1, and S2. Underlined regions indicate either the 5'-handle of the unprocessed/cas6-processed crRNA or the 3'-antitag of the target RNA. Bold in DTR (Deoxyribonucleotide target RNA) highlights Csm3-mediated ribonucleotide cleavage sites replaced by deoxyribonucleotides.

Nucleic acid	Sequence	Source
Repeat encoded by pACYC plasmids	5′aaauacaaccgcuccucgauaaaaggggacgagaac 3′	
Spacer encoded by pACYC plasmids	5′AUACGUUCUUUGAACCAAGCUUCAACUCC 3′	
HDV ribozyme	5′GCCGGCCAUGGUCCCAGCCUCCUCGCUGGCGGCCGGUGGGCAACAUUC CGAGGGGACCGUCCCCUCGGUAAUGGCGAAUGGGAC 3′	
37mer mature crRNA	5′acgagaacAUACGUUCUUUGAACCAAGCUUCAACUCC 3′	
43mer mature crRNA	5′acgagaacAUACGUUCUUUGAACCAAGCUUCAACUCCGCCGGC 3′	
NTR used in activity assays	3′ugcucuugUAUGCAAGAAACUUGGUUCGAAGUUGAGG 5′	IDT
CTR used in activity assays	3′acgagaacUAUGCAAGAAACUUGGUUCGAAGUUGAGG 5′	IDT
PTR used in activity assays	3′UAUGCAAGAAACUUGGUUCGAAGUUGAGG 5′	IDT
ATR used in activity assays	3′GGAGUUGAAGCUUGGUUCAAAGAACGUAU 5′	IDT
DTR used in activity assays	3′acgagaacUAUGC**A**AGAAA**C**UUGGU**T**CGAAG**T**UGAGG 5′	IDT
PolyA(NTR): Target RNA used in Csm-NTR complex	3′AAAAAAugcucuugUAUGCAAGAAACUUGGUUCGAAGUUGAGGAA 5′	IDT
PolyA(CTR): Target RNA used in Csm-CTR complex	3′AAAAAAacgagaacUAUGCAAGAAACUUGGUUCGAAGUUGAGGAA 5′	IDT
Fluorescence DNA Reporter	5′/5Alex594N/TTATTATT/3lAbRQSp/3′	IDT
Fluorescence RNA Reporter	5′/56-FAM/rArArArArA/3lABkFQ/3′	IDT
5Cy3-CTR	5′/5Cy3/AAGGAGUUGAAGCUUGGUUCAAAGAACGUAUcaagagcaAAA AAA 3′	IDT

M13mp18	https://www.snapgene.com/resources/plasmid-files/?set=basic_cloning_vectors&plasmid=M13mp18	New England Biolabs

Table S4: Amino acid sequences of *Lactococcus lactis* CRISPR-Cas (Csm) system. Related to Figures 1, 2, 3 and 4 and S1.

Subunit	Amino acid sequence
Cas10/Csm1	MDKINLVCGSLLHDIGKIIYRGTSERAKHSKLGGDFIKSFEQFRNTELTDCIRYH<br>HAQEITSVKSNKEKNSLFYITYIADNISSGMDRRKDLEEGAEGFNWDKKVALGSV<br>FNVLNEKEKGRQNYSYPFVARTRIKEEPLNFPTATQNQYTTSYYDGLITDMKTIL<br>QRLKPDKEHINSLLQMMESLWSYVPSSTDKNQLVDISLYDHSRTTAAIASAIYDY<br>FQAENITDYQKELFDYNATEFYDKNAFLMMNFDMSGVQNFIYNISGSKALKSLRA<br>RSFYLDMLLEYISDNLLEKLELSRANILYVGGGHAYLLLANTNKTKAILSDFEHD<br>LKTWFLDKFKIDLYVAMAYTEVSANDLMNHNGHYRDIYRRLSQKTSAKKANRYTA<br>EEILNLNHQGTENARECRECKRSDLLIEEDDICEICDSLQKVSRDLTRENIFVIA<br>NEGVLDMPFGKKMSALSYSQADKLKKSNAEVQIYAKNISEIGQNLMTRIDMGDYT<br>YRSDFHEMLEEVEVGINRLGVLRADVDNLGQAFINGIPDDYLSISRTATFSRAMS<br>RFFKNYLNQLLAEKSYKINVIYAGGDDLFMIGAWQDILDFSIVLKQKFADFTQNK<br>LSISAGIGMFREKYPVARMASLTGDLEDAAKDYKPDERAVQATKNAVTLFDATNV<br>FSWDTLENDIFVKLDAITKNFEKLDETGKAFIYRLIDLLRGVNENQQINIARLAY<br>TLSRMEEKIGKTFAQELYNWANADRKTLIMALEIYILKTRER
Csm2-NHis	MGHHHHHHSGGTELKIGNEKVNSTNFGDFAEKAIRGINHKPFVNSKGGEQKITTS<br>KIRGILELVNKVYNRVINTNDVELSENILADIAYIKVKIAYESGREPVVKDFIQR<br>TAFTAAITDVMNQRTRESFLLFARYVESLIAYFKFYGGKD
Csm3	MKLVIEGTIVLKTGMHIGGSSDFSAIGAVDSPVVRDTLTRLPLIPGSSLKGKMRY<br>LLAKELNNGILLNEPNNDQDEILRLFGSSEKDKIRRARLKFNDIKLSNLAELETF<br>NVSSTEVKFENTINRKTAVANPRQIERVIAGSKFDFEIFYNLDDIKEVEKDFENI<br>KQGFDLLEFDYLGGHGTRGSGRIAFENLSVITAVGNFEKINTLNEILGA
Csm4	MKIIKLYFESPVHFGEKRLSESKITFSADTLFSALMIEAVGLGKEDEFYQLASNN<br>LVKFSDAFPFIDQYYYIPKPMFNLKLEKEDENPSKAFKKLLYVPIDSLEDYLSGG<br>LDAYFERESFNLGKLALSEKVQQHDFKDSEPYNVGTFTFKENTGLYVLIEQTHPL<br>LEELLENLQYSGIGGKRNSGYGKFKFEILEDSDIEDLFSAKGNRKILLSGALPKD

	AELEQALKNASYLLERRGGFVQSDTYATNLVKKQDLYVFKSGSTFENSFDGDIYQ VGKKGNHPVYKYAKSFFLEVSV
Csm5	MKKTYRVTLTALGPIFIGGGEKLKKYEYIFDKQKKVAHMIDHTKFTKYLLEKNLL DDFTSRVNSHFDLYDYLVNKKGIVFMPLVKYSVPVAQFRTEVKNRFGKPISSPPM NDLNTFVKDAFGRPYIPGSSLKGALRTAILNDLKEDTKENEVFAHLQVSDSETID LENLKVYQKVDYSKTAKPLPLYRECLKPNTEITFTVSFDDEYLTLKKIQNALHKT YQHYYIKWLKGGKVGETLIKGVYDSHADELKKNTFALDQPSQNQGEIIYIGGGAG FVSKTLHYKSKNRDQARNDSFDILKQLFRTTYSKMRSVPDNVPVALKLAVETKTF NGRVTGKHYLEMGKARIKLEELK
Csm6	MKILISAVGDTDPIRNFHDGPLLHIVRVYRPEKIVLVHSERSLTKHDKLVKALKS IKDYSPEIIQDGVVLPDAQVAIFDEMYDTVSSIVKKYISDDEIILNISSATPQII SAMFAVNRISDFNVTAVQVKTPQHKSNEGLRHDNQEDIDKLIETNLDNQSDYENR TLADTGMKFSQDLTKRNLKALIDNYDYQGALELLKKQKSFSNIKELRKKLTEISD TIKIQGMPDKIVKSKLSNQAKSALNSYLNIDRNHKQGNIAEVLIRVKSLVEFILE DYLNNHFLDVITYKDGKPFLNASKYPEILKKFQEDAEMRGKEYHSGYLSLPAYIG ILKFFEPNHDLLKHIYKIQEINQDRNKVAHSLQAFDRKNLKKVSSAVFASKQILL ASFDIDNHWFSFYEDLNQEIKKLL
Cas6	MIVKLRYKINLPNSLRTQNIGSTLHGVLMELLPSELVEHLHNLSYNPFRQRLIFE KELVIWEIVGLHKMVSEELLKLENLREITIKRAQKTVSLSLLSKDAIAVDDLVKK EMGREIDSRIISLKFTSPTSFKANGHYDIFPDIRKIFRSLMMNFDFFSETTKIYD YEVLSYIEENVHIVSYKLMTKNFHLEKIKVKGFQGDMTLKVTGAEQFVKLVLLMI KYATFAGIGMKTSLGMGGVSINERHYLR

Table S5. List of cross-linked residues and comparison of cross-linking abundance in CTR and NTR complexes

Cross-linked residues	Peptide identified in 1st subunit	Peptide identified in 2nd subunit	Abundance ratio CTR/NTR	Abundance ratio NTR/CTR
726_1-149_2	IGKTFAQELYNWANADR	FYGGKD	0.86	1.46
683_1-149_2	NFEKLDETGK	FYGGKD	1.54	1.39
723_1-126_3	MEEKIGK	KTAVANPR	0.96	1.32
683_1-126_3	NFEKLDETGK	KTAVANPR	1.20	Not observed
683_1-86_3	NFEKLDETGK	LFGSSEKDK	0.58	1.83
374_1-83_4	LSQKTSAK	LEKEDENPSK	0.90	0.90
379_1-83_4	KANR	LEKEDENPSK	0.87	1.15
46_2-126_3	GINHKPFVNSKGGEQK	KTAVANPR	0.97	1.02
56_2-126_3	ITTSKIR	KTAVANPR	Not observed	1.22
149_2-309_5	FYGGKD	TTYSKMR	1.32	1.27
86_3-279_4	LFGSSEKDK	KGNHPVYK	7.14	0.22
86_3-137_4	LFGSSEKDK	VQQHDFKDSEPYNVGTFTFK	2.75	0.30
126_3-144_5	TAILNDIKEDTK	KTAVANPR	1.12	1.12
126_3-300_5	NDSFDIKKQLFR	KTAVANPR	1.12	1.45
618_1-271_1	EKYPVAR	ALKSLR	1.14	1.14
135_1-597_1	IKEEPLNFPTATQNQYTTSYYDGLITDMK	QKFADFTQNK	0.62	1.63
321_1-374_1	TKAILSDFEHDLK	LSQKTSAK	1.43	1.16
105_2-149_2	IAYESGREPVVKDFIQR	FYGGKD	1.09	1.43
56_2-149_2	FYGGKD	FYGGKD	1.05	1.19
86_3-126_3	LFGSSEKDK	KTAVANPR	1.04	1.56
88_3-126_3	LFGSSEKDKIR	KTAVANPR	1.06	1.56
52_5-75_5	YLLEKNLLDDFTSR	KGIVFMPLVK	1.04	0.90
337_5-98_5	VTGKHYLEMGK	TEVKNR	0.96	0.98
182_5-309_5	TAKPLPLYR	TTYSKMR	1.38	1.30

Table S6. KEY RESOURCES TABLE

Reagent/Resource	Source	Identifier
**Bacterial Strains**		
E. coli DH5a	ATCC	67879
E. coli NiCo(DE3)	New England BioLabs	C2529H
E. coli BL21 AI	Invitrogen	C6070-03
E. coli TOP10	Invitrogen	C404010
**Reagents and Chemicals**		
LB Broth, Miller	VWR	J106-2KG
Terrific broth	Sigma	T 0918
Chloramphenicol	Sigma	C-0378
Agar, bacteriological	VWR	J637-1KG
Agarose	Fisher BioReagents	BP160-500
Isopropyl-β-D-Thiogalactopyranoside (IPTG)	Affymetrix	367-93-1
Sodium chloride	Sigma	S7653
(4-(2-hydroxyethyl)-1-piperazineethanesulfonic acid) (HEPES) sodium salt	Sigma	H7006-500G
Imidazole	ACROS Organics	288-32-4
Glycerol	Fisher Chemical	G33-4
2-Mercapotethanol	VWR	M131-250mL
Magnesium chloride	Sigma	M8266-100G
Manganese chloride	Sigma	M3634-100G
Nickel(II) sulfate hexahydrate	Sigma	227676-100G
Sodium dodecyl sulphate	VWR	0227-100G
4-Morpholinepropanesulfonic acid (MOPS)	Sigma	M1254-250G
Urea	Fisher Biotech	BP169-212
Acrylamide/Bis-acrylamide, 29:1	Sigma	A2792-100ML
Tetramethylethylenediamine (TEMED)	OmniPur	110-18-9
Ammonium Peroxydisulfate	Fisher Chemical	A682-500
Bromophenol Blue	Bio-Rad	161-040
PageRuler Prestained Protein Ladder	ThermoFisher Scientific	26616
Gel loading dye, Purple	New England Biolabs	B7025S
Dithiothreitol (DTT)	VWR	0281-25G
M13mp18	New England Biolabs	N4040S
KLD Enzyme Mix 25 rxns	New England Biolabs	M0554S
BamHI	New England Biolabs	R0136S

EcoRI	New England Biolabs	R0101S
T4 DNA Ligase	New England Biolabs	M0202L
Acetic acid, Glacial	EMD Millipore Corporation	AX0073.9
Methanol	Spectrum Chemical Mfg. Corp.	M1240
SYBR Gold Nucleic Acid Gel Stain	Invitrogen	S11494
[α-^{32}P] ATP	PerkinElmer	BLU002Z250UC
Ethidium bromide	Sigma	E-7637
**Commercial Kits**		
E.Z.N.A. Plasmid DNA Miniprep Kit	Omega BIO-TEK	D6942-01
ZymoPURE II Plasmid Midiprep Kit	Zymo Research	D4201
Silver Stain Plus Kit	Bio-Rad	1610449
Q5 Site-directed Mutagenesis Kit	New England Biolabs	E0554S
Gibson Assembly Cloning Kit	New England Biolabs	E5510S
**Deposited data**		
Binary/Apo LICsm complex	This study	PDB 6XN5
LICsm-NTR complex	This study	PDB 6XN7
LICsm-CTR_43 complex	This study	PDB 6XN3
LICsm-CTR_32 complex	This study	PDB 6XN4
**Software**		
Adobe Illustrator	https://www.adobe.com/products/illustrator.html	
The PyMOL Molecular Graphics System	https://pymol.org/2/	
COOT	https://www2.mrc-lmb.cam.ac.uk/personal/pemsley/coot	
Chimera X	https://www.cgl.ucsf.edu/chimerax/	
Chimera	https://www.cgl.ucsf.edu/chimera/	
Phenix	https://www.phenix-online.org/	
RELION 3.0/3.1	https://github.com/3dem/relion	
CryoSPARC	https://cryosparc.com/	
CisTEM	https://cistem.org/	
Motion cor2	https://emcore.ucsf.edu/ucsf-motioncor2	
Gctf	https://www2.mrc-lmb.cam.ac.uk/research/locally-developed-software/zhang-software/	
SnapGene Viewer	https://www.snapgene.com/	
T-Coffee	http://tcoffee.crg.cat/apps/tcoffee/do:regular	

Boxshade	http://www.ch.embnet.org/software/BOX_form.html

Supplementary References:

1. J. Zivanov *et al.*, New tools for automated high-resolution cryo-EM structure determination in RELION-3. *eLife* **7** (2018).

2. A. Punjani, J. L. Rubinstein, D. J. Fleet, M. A. Brubaker, cryoSPARC: algorithms for rapid unsupervised cryo-EM structure determination. *Nature methods* **14**, 290-296 (2017).

3. T. Grant, A. Rohou, N. Grigorieff, cisTEM, user-friendly software for single-particle image processing. *eLife* **7** (2018).

4. J. Zivanov, T. Nakane, S. H. W. Scheres, Estimation of high-order aberrations and anisotropic magnification from cryo-EM data sets in RELION-3.1. *IUCrJ* **7**, 253-267 (2020).

5. E. F. Pettersen *et al.*, UCSF Chimera--a visualization system for exploratory research and analysis. *J Comput Chem* **25**, 1605-1612 (2004).

6. T. D. Goddard *et al.*, UCSF ChimeraX: Meeting modern challenges in visualization and analysis. *Protein Sci* **27**, 14-25 (2018).

7. P. Emsley, B. Lohkamp, W. G. Scott, K. Cowtan, Features and development of Coot. *Acta crystallographica. Section D, Biological crystallography* **66**, 486-501 (2010).
