# Supplementary material for: Structural and biochemical characterization of in vivo assembled Lactococcus lactis CRISPR-Csm complex
Source: Commun Biol. 2022 Mar 29;5:279. doi: 10.1038/s42003-022-03187-1 (PMC8964682; doi:10.1038/s42003-022-03187-1)
Supplement: Supplementary file 2 — Reporting Summary [file 42003_2022_3187_MOESM2_ESM.pdf]

nature portfolio

Corresponding author(s): Hong Li

Last updated by author(s): Nov 30, 2021

# Reporting Summary

Nature Portfolio wishes to improve the reproducibility of the work that we publish. This form provides structure for consistency and transparency in reporting. For further information on Nature Portfolio policies, see our Editorial Policies and the Editorial Policy Checklist.

## Statistics

For all statistical analyses, confirm that the following items are present in the figure legend, table legend, main text, or Methods section.

| n/a | Confirmed | |
|---|---|---|
| ☐ | ☒ | The exact sample size (*n*) for each experimental group/condition, given as a discrete number and unit of measurement |
| ☐ | ☒ | A statement on whether measurements were taken from distinct samples or whether the same sample was measured repeatedly |
| ☒ | ☐ | The statistical test(s) used AND whether they are one- or two-sided<br>*Only common tests should be described solely by name; describe more complex techniques in the Methods section.* |
| ☒ | ☐ | A description of all covariates tested |
| ☒ | ☐ | A description of any assumptions or corrections, such as tests of normality and adjustment for multiple comparisons |
| ☒ | ☐ | A full description of the statistical parameters including central tendency (e.g. means) or other basic estimates (e.g. regression coefficient) AND variation (e.g. standard deviation) or associated estimates of uncertainty (e.g. confidence intervals) |
| ☒ | ☐ | For null hypothesis testing, the test statistic (e.g. *F*, *t*, *r*) with confidence intervals, effect sizes, degrees of freedom and *P* value noted<br>*Give P values as exact values whenever suitable.* |
| ☒ | ☐ | For Bayesian analysis, information on the choice of priors and Markov chain Monte Carlo settings |
| ☒ | ☐ | For hierarchical and complex designs, identification of the appropriate level for tests and full reporting of outcomes |
| ☒ | ☐ | Estimates of effect sizes (e.g. Cohen's *d*, Pearson's *r*), indicating how they were calculated |

*Our web collection on statistics for biologists contains articles on many of the points above.*

## Software and code

Policy information about availability of computer code

| | |
|---|---|
| Data collection | Cryo-EM data collection was performed at Biological Science Imaging Resource (BSIR), Florida State University using FEI Titan Krios equipped with Gatan Bioquantum K3 direct electron detector (ThermoFisher Scientific). |
| Data analysis | Cryo-EM data image processing and data analysis softwares include Relion3, Relion 3.1, CryoSparc, MotionCorr2, Cistem. Structures are refined by the program suite Phenix , modeled and visualization with COOT ( MRC LMB , https://www2.mrc-lmb.cam.ac.uk/personal/pemsley/coot ), PyMOL ( The PyMOL Molecular Graphics System , https://pymol.org/2/ ) or Chimera (https://www.cgl.ucsf.edu/chimera/). All the figures are generated by Adobe illustrator ( https://www.adobe.com/products/illustrator.html). No custom algorithms or softwares were used. |

For manuscripts utilizing custom algorithms or software that are central to the research but not yet described in published literature, software must be made available to editors and reviewers. We strongly encourage code deposition in a community repository (e.g. GitHub). See the Nature Portfolio guidelines for submitting code & software for further information.

## Data

Policy information about availability of data

All manuscripts must include a data availability statement. This statement should provide the following information, where applicable:

- Accession codes, unique identifiers, or web links for publicly available datasets
- A description of any restrictions on data availability
- For clinical datasets or third party data, please ensure that the statement adheres to our policy

*Provide your data availability statement here.*

# Field-specific reporting

Please select the one below that is the best fit for your research. If you are not sure, read the appropriate sections before making your selection.

☒ Life sciences ☐ Behavioural & social sciences ☐ Ecological, evolutionary & environmental sciences

For a reference copy of the document with all sections, see nature.com/documents/nr-reporting-summary-flat.pdf

# Life sciences study design

All studies must disclose on these points even when the disclosure is negative.

| | |
|---|---|
| Sample size | A total of 5179 raw images (CTR-bound complex), 4382 raw images (Apo complex) and 3922 raw images (NTR complex) were collected. |
| Data exclusions | 2636 images (CTR-bound complex), 2046 images (Apo complex) and 2030 images (NTR complex) were rejected due to bad ice, astigmatism, drift and/or poor sample quality. |
| Replication | All in vitro biochemical assays and in vivo functionality assays were performed as triplicates for consistency. |
| Randomization | n/a |
| Blinding | n/a |

# Reporting for specific materials, systems and methods

We require information from authors about some types of materials, experimental systems and methods used in many studies. Here, indicate whether each material, system or method listed is relevant to your study. If you are not sure if a list item applies to your research, read the appropriate section before selecting a response.

## Materials & experimental systems

| n/a | Involved in the study | |
|---|---|---|
| ☒ | ☐ | Antibodies |
| ☒ | ☐ | Eukaryotic cell lines |
| ☒ | ☐ | Palaeontology and archaeology |
| ☒ | ☐ | Animals and other organisms |
| ☒ | ☐ | Human research participants |
| ☒ | ☐ | Clinical data |
| ☒ | ☐ | Dual use research of concern |

## Methods

| n/a | Involved in the study | |
|---|---|---|
| ☒ | ☐ | ChIP-seq |
| ☒ | ☐ | Flow cytometry |
| ☒ | ☐ | MRI-based neuroimaging |
